# Discovery of MBL-AB01: a novel antibacterial xanthone antibiotic with high activity against methicillin-resistant *Staphylococcus aureus*

**DOI:** 10.1128/aem.01346-25

**Published:** 2025-12-08

**Authors:** Kristin Fløgstad Degnes, Anna Nordborg, Giang-Son Nguyen, Guro Kruge Nærdal, Tonje Marita Bjerkan Heggeset, Peter Molesworth, Sigrid Hakvåg, Randi Aune, Vu To Nakstad, Johan Evenäs, Klara Jonasson, Trond Erling Ellingsen, Alexander Wentzel, Geir Klinkenberg, Håvard Sletta

**Affiliations:** 1 SINTEF Industry, Biotechnology and Nanomedicine 275243, Trondheim, Norway; 2 Department of Biotechnology and Food Science, NTNU Norwegian University of Science and Technology 205785, Trondheim, Norway; 3 RG Discovery AB, Lund, Sweden; Centers for Disease Control and Prevention, Atlanta, Georgia, USA

**Keywords:** *Staphylococcus aureus*, MRSA, xanthones, drug discovery, minimum inhibitory concentration, biosynthetic gene cluster, bioreactor

## Abstract

A novel compound denoted MBL-AB01 was isolated from a marine *Actinoalloteichus*, which belongs to a rare and underexplored class of *Actinobacteria*. This work demonstrates that the novel compound MBL-AB01 shows very high activity *in vitro* against six methicillin-resistant *Staphylococcus aureus* strains, and high activity against a panel of three other Gram-positive strains, including a vancomycin-resistant *Enterococcus faecium*. Structure elucidation of the compound revealed that MBL-AB01 is a polycyclic xanthone antibiotic closely related to the bioactive compounds: xantholipin and lysolipin. This class of antibiotics has caught interest due to its unique chemical structure and diverse biological activity. The gene cluster encoding MBL-AB01 production was identified, and the individual genes within the cluster were annotated along with proposed functional roles. The compound was produced by bioreactor fermentations, and significantly higher yields of MBL-AB01 were obtained after classical mutagenesis and fermentation process improvements.

**IMPORTANCE:**

Methicillin-resistant *Staphylococcus aureus* (MRSA) infections have become a great challenge in hospitals over the last decades, and MRSA is currently one of the six pathogens on the World Health Organization priority list. Here, we demonstrate that the novel antibiotic MBL-AB01 has excellent antibacterial properties against six *S. aureus* strains, including MRSA. MBL-AB01 belongs to the poorly explored class of polycyclic xanthones, thereby fulfilling innovation criteria for the development of new antibiotics. The compound can be produced in sufficient amounts for early formulation development and pre-clinical trials.

## INTRODUCTION

The rapid rise and spread of antibiotic-resistant pathogenic bacteria is alarming, and the burden of antimicrobial resistance poses significant impact on society and well-being (1). Methicillin‐resistant *Staphylococcus aureus* (MRSA) remains at the core of this threat and is one of the pathogens on the World Health Organization (WHO) priority list (2). MRSA strains are resistant to all β-lactam antibiotics except ceftaroline and ceftobiprole (3–5). They also frequently show resistance to other antibiotics such as erythromycin, ciprofloxacin, gentamicin, and amikacin. Consequently, the primary treatment options for MRSA infections are currently vancomycin, a glycopeptide, and daptomycin, a cyclic lipopeptide (6). However, resistance has been observed against essentially all classes of compounds, including the glycopeptide antibiotics (7). Therefore, there is a pressing need to discover new antimicrobial compounds from less explored antibiotic groups. Most of the antibiotics in clinical use today originate from soil-derived *Actinobacteria*, particularly the *Streptomycetes*. The main groups of non-*Streptomyces* species that have been reported to produce antibacterial compounds are *Micromonospora*, *Nocardia*, *Actinomadura*, *Actinoplanes*, *Streptoverticillium,* and *Saccharopolyspora* (8).

Polycyclic xanthone derivatives are an expanding group of structurally complex aromatic compounds typically originating from a single C26 or C28 polyketide chain (9). Bioactive xanthones are produced by a range of different *Actinobacteria* isolated from both soil and marine environments (10). However, the marine environment is still an underexplored source of xanthones, and by 2022, less than 50 complex xanthones of marine origin had been isolated (11). The bioactive xanthone antibiotics, xantholipin (12) and lysolipin (13), are both halogenated with chlorine, and their structures have low hydrogen/carbon ratios. Buanmycin (14) and the recently discovered sattahipmycin (15) are not halogenated, but show high structural similarity to xantholipin and lysolipin. Xantholipin was isolated from a *Streptomyces* sp. found in a soil sample in China, sattahipmycin was isolated from a *Streptomyces* sp. found in a sediment sample collected in the Gulf of Thailand, and buanmycin was isolated from a *Streptomyces* sp. harvested from a marine mud sample in Korea.

In recent years, the search for new bioactive compounds has moved toward the more underexploited habitats such as the marine environment (8, 16). Between 2003 and 2006, as part of a bioprospecting campaign, we conducted a screening effort to identify new bioactive compounds produced by marine Actinomycetes in the Trondheim fjord, Norway. Our findings demonstrated that many of the isolates belonged to other classes of *Actinobacteria* than the *Streptomyces* species (17). One bacterial isolate discovered in this screening program was the sea-water-dependent MP127-IG17 (also referred to as TSI127-17 ID by Engelhardt [17]) isolated from a marine sponge at 60 m depth. Molecular taxonomy, based on 16S rRNA gene similarity, revealed that the closest relative to MP127-IG17 is the rare Actinobacteria *Actinoalloteichus hymeniacidonis* HPA177, which shares 98% gene similarity (17). *Actinoalloteichus hymeniacidonis* HPA177 was initially isolated from a marine sponge found on a beach in China (18). Subsequently, a Danish research group isolated and characterized a macrolactam xanthone antibiotic, named Xanthobaccin A, from this strain (19).

Agar diffusion assays previously performed by our group revealed that extracts from MP127-IG17 were active against the Gram-positive bacterium *Micrococcus luteus*, the vancomycin-resistant *Enterococcus faecium,* and the fungus *Candida albicans* (17). Here, we describe the discovery of a novel antibacterial xanthone purified from this strain. This compound has high potential as a drug candidate based on its activity against MRSA and low toxicity in human cells. Additionally, we demonstrate that the compound can be produced and purified in sufficient amounts for pre-clinical studies.

## RESULTS

### Discovery of the active compound produced by MP127-IG17

The rare group of *Actinobacteria* to which MP127-IG17 belongs, and its previously described bioactivity against the vancomycin-resistant strain *E. faecium* (17), encouraged the selection of the isolate for the studies described below. Based on previous data (17), one medium (PM6) was selected for production in shake flasks. Dimethyl sulfoxide (DMSO) extracts of the broth confirmed bioactivity against *M. luteus* ATCC 9341, vancomycin-resistant *E. faecium* CCUG 37832, and *C. albicans* CCUG 39343 in the liquid-based bioassay (Table S1).

High-pressure liquid chromatography (HPLC) fractionation of the active DMSO extract, followed by activity assay of the fractions against *M. luteus* ATCC9341 and *E. faecium* CCUG37832, showed that the bioactivity could be traced back to one HPLC fraction. By liquid chromatography-diode array detector-time of flight high-resolution MS (LC-DAD-QTOF) analysis of the active fraction, it was found that a UV absorption peak with UV maxima at 395 nm correlated with peaks in LC-QTOF chromatograms. The monoisotopic mass of the compound was 551.062 Da, and the compound was named MBL-AB01.

### Improvement of the MBL-AB01 yield

**Fig 1:**
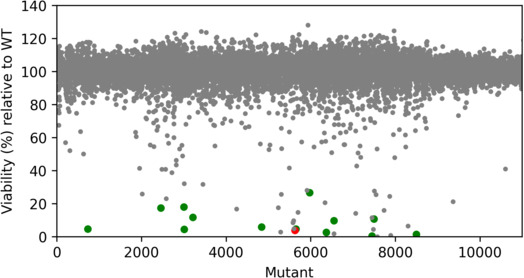
Growth of *E. faecium* CTC492E after exposure to DMSO extracts of the MP127-IG17 mutants obtained by UV mutagenesis. Totally, 10,752 assays with extracts from mutants and 394 assays with extracts of the WT control (not shown) were analyzed. Gray scatters represent mutants that were not selected for further analysis, large green scatter represents mutants selected for shake flask cultivation, and the large red scatter represents the mutant chosen after evaluation in shake flasks.

**Fig 2:**
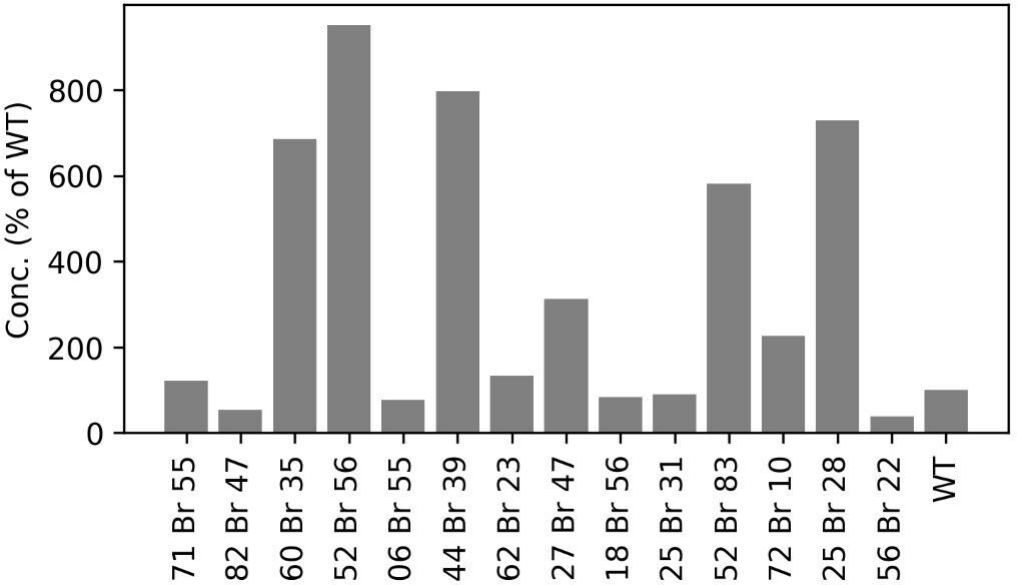
Relative concentration of MBL-AB01 in culture extracts from 14 mutant candidates, expressed as a percentage of the wild-type (WT) isolate. The mutants were selected based on a viability screen of 10,752 strains and cultivated in shake flasks. The highest yield (ninefold greater than the WT) was observed in mutant MP127-IG17(Pl52Br56/E8).

Low titers of MBL-AB01 necessitated yield improvements for further purification and characterization. In the initial phase, a purified analytical standard of MBL-AB01 was not available. Therefore, the UV absorption peak at 395 nm, corresponding to MBL-AB01, was used as a relative indicator of yield. First, the yield of MBL-AB01 was improved by replacing the 10 g/L glucose in the PML6 medium with 30 g/L soluble starch (PML6_MOD3 medium, data not shown). To further increase the yield of MBL-AB01, UV mutagenesis of MP127-IG17 was performed. Robotic picking of the surviving colony-forming spores after 60 seconds (98% killing rate) and 70 seconds of UV exposure resulted in a library of 10,752 isolates. After cultivation and DMSO extraction of the library, dilutions of the extracts were tested for activity against *E. faecium* CTC492. At the selected dilution, wild-type extracts did not inhibit the growth of the indicator organism, but several extracts of the library isolates did (Fig. 1). In total, 14 candidates were selected for a second evaluation in shake flasks using PML6_MOD3 medium, and one candidate (MP127-IG17(Pl52Br56/E8) produced 9 times more MBL-AB01 than the wild-type strain (Fig. 2).

Purified material was needed to determine the concentration of MBL-AB01 in the culture extracts of MBL-AB01. Thus, MP127-17 PI52Br56 was selected for upscale production with controlled pH, oxygen, temperature, and agitation in PML6_MOD3 medium. The compound was purified by preparative HPLC to obtain material for analytical standards, NMR structure determination, and bioactivity assays. HPLC separation was performed under slightly alkaline conditions obtained by supplementing the aqueous mobile phase with ammonia. To improve the stability of the compound, the pH of the HPLC fractions was reduced prior to further work-up. Purified MBL-AB01 appeared as an amorphous yellow powder.

**Fig 3:**
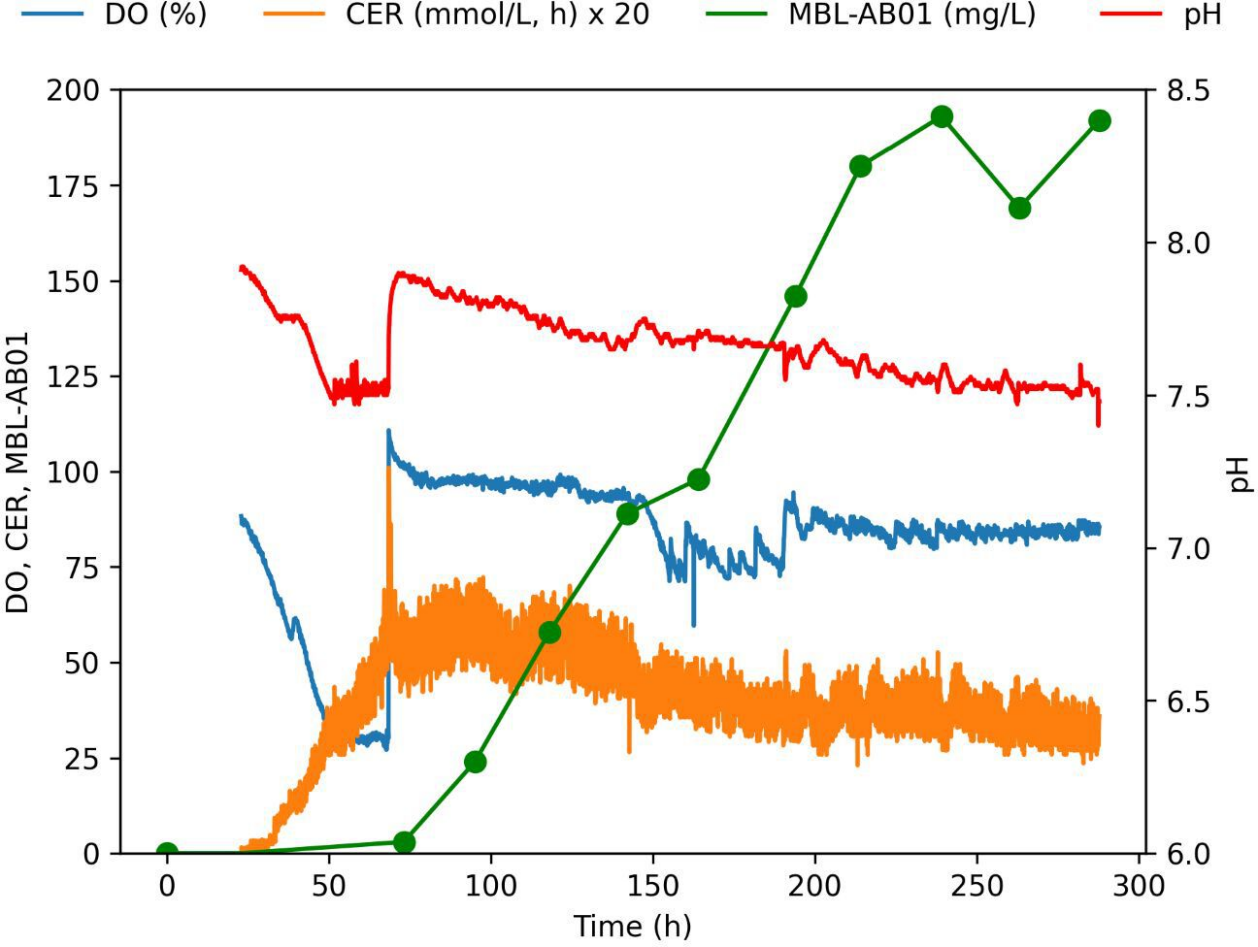
Key parameters monitored during bioreactor cultivation of mutant MP127-IG17 Pl 52 BR 56 for the production of MBL-AB01. The cultivation was performed in 3 L controlled bioreactors using PML6_MOD3 medium. DO, CER, and pH were continuously monitored, whereas the concentration of MBL-AB01 was measured at 10 sampling points. Dissolved oxygen (DO) was measured as percent of the oxygen concentration in oxygen-saturated water at 25°C, and carbon evolution rate (CER) was measured as mmol CO_2_/L, h in the exhaust gas from the bioreactor.

**Fig 4:**
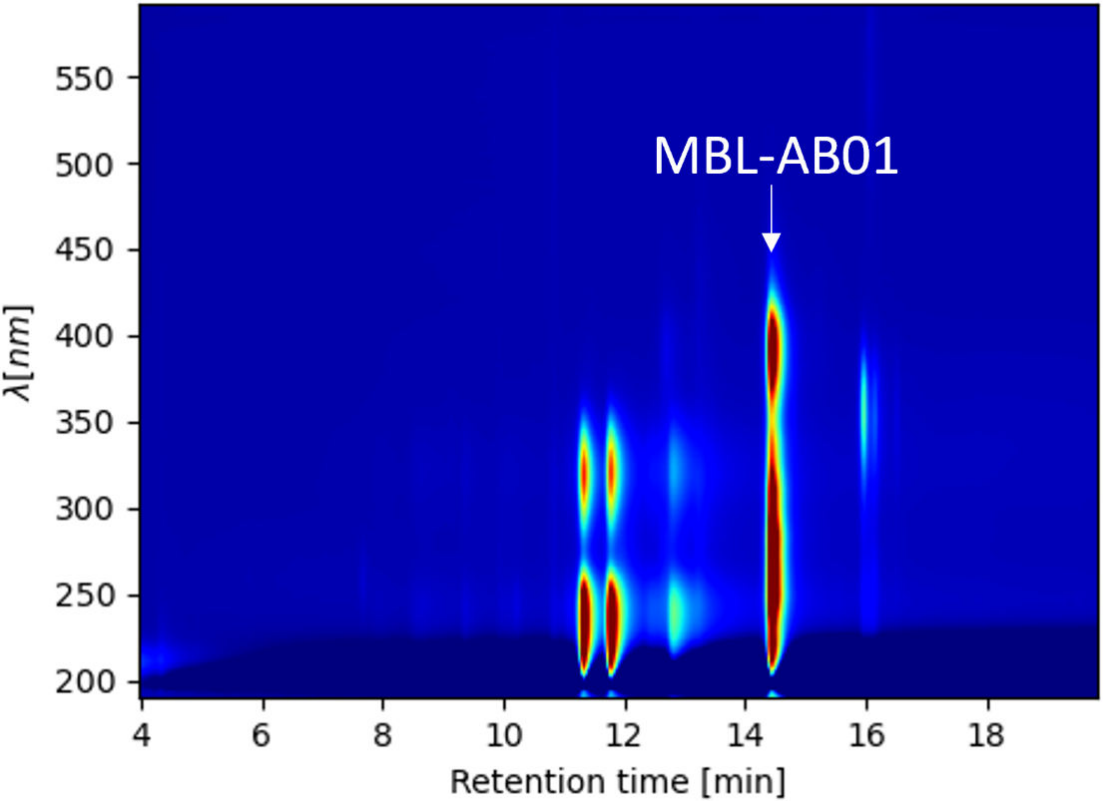
LC-DAD isoplot of DMSO extracted broth after cultivation in PML6_MOD3 medium in a 3 L bioreactor.

The concentration of nitrogen sources in the PML6_MOD3 medium was relatively low (0.5 g nitrogen/L). Furthermore, a combination of soluble starch from the supplier Difco and glutamate as carbon- and nitrogen sources, respectively, has previously proved successful for secondary metabolite productions using *Actinobacteria* (20). Thus, the effects of varying and increasing the nitrogen sources and increasing the concentrations of peptone and yeast extract were evaluated in a medium screening study in 3 L bioreactors (one fermentation for each condition), using the selected mutant strain. In addition, pH regulation and various stirring speeds (to ensure higher oxygen transfer and higher shear forces) were tested. The highest volumetric yield of MBL-AB01 was obtained in PML6_MOD3 with the pH controlled at 7.5. This condition yielded 193 mg/L MBL-AB01 (Table 1; Fig. 3). The addition of Na-glutamate, NaNO_3_, or extra yeast extract also improved production compared to the corresponding reference cultivations (PML6_MOD5). In contrast, supplementing the medium with NH_4_Cl reduced the yield, and the addition of soy flour completely inhibited the production. The strain grew very poorly with low respiration and production of MBL-AB01 in a medium without artificial seawater. Analyses of the extracted broth with LC-DAD-QTOF showed that UV-absorbing impurities were eluting both before and after MBL-AB01, and that the different media supplements impacted the impurity profile of the broth. It was observed that both glutamate and NaNO_3_ supplements increased the ratio of the impurity eluting at 15.8 min in the LC-chromatogram. An example of an LC-chromatogram is shown in Fig. 4.

**TABLE 1:** Volumetric yields of MBL-AB01 in cultivations of the mutant strain MP127-IG17 Pl 52 Br 56 in 3-L bioreactors^*b*^

Media	Yield (mg/L)	Supplements^*a*^	Supplier of starch	pH control	Oxygen control	Stirring speed
PML6_MOD3	193		Sigma	pH = 7.5		1,500 rpm
PML6_MOD5	38		Difco		30%	
PML6_MOD6	65	Glut.	Difco	MOPS	30%	
PML6_MOD7	21	NH4Cl	Difco	MOPS	30%	
PML6_MOD8	87	NaNO3	Difco	MOPS	30%	
PML6_MOD9	94		Difco	pH = 7.5	30%	
PML6_MOD10	72	YE	Difco	pH = 7.5	30%	
PML6_MOD11	0	Soy flour	Difco	pH = 7.5	30%	
PML6_MOD12	23		Difco		30%	
PML6_MOD16	72	YE, peptone, CaCO_3_	Sigma	pH = 7.5		1,500 rpm
PML6_MOD17	84	Cornsteep	Difco	pH = 7.5		1,500 rpm

^*a*^ Glut: glutamate, YE: yeast extract.

^*b*^ Carbon and nitrogen sources, two different brands of starch, pH, control, and shear forces were parameters that were tested. High shear forces were obtained by increasing the stirring speed to 1,500 rpm. In the remaining bioreactors, the dissolved oxygen concentration was controlled at a minimum of 30% of saturation by controlling the stirring speed.

### *In vitro* antibacterial and cytotoxic properties of MBL-AB01

Purified MBL-AB01 was tested against a panel of Gram-negative and Gram-positive pathogens. The compound had no detected activity against the Gram-negative panel within the tested concentration range (data not shown). However, it showed strong activity against both *M. luteus* with a MIC of 0.06 µg/mL, as well as six *S. aureus* isolates, including MRSA, all of which had MIC ≤0.03 µg/mL. MBL-AB01 also inhibited the growth of vancomycin-resistant bacterial strains represented by *E. faecium* CCUG 37832 and *E. faecium* CTC 492, with an MIC of 0.25 and 0.5 µg/mL, respectively (Table 2). *In vitro* cytotoxicity after exposure to MBL-AB01 was tested against one non-transformed cell line (IMR-90 human lung fibroblast). Toxic effects were observed at concentrations of 50 µg/mL MBL-AB01 and above. Anticancer activity was tested against five human cancer cell lines. The half-maximal effective concentration (EC_50_) values were >20 µg/mL for all these cell lines (Fig. S1).

**TABLE 2:** *In vitro* antibacterial activity (MIC, µg/mL) of MBL-AB01, vancomycin, gentamicin, and streptomycin against Gram-positive bacterial pathogens^*a*^.

	MBL-AB01	Vancomycin	Gentamicin	Streptomycin
*E. faecium* CCUG 37832	0.25	>16	16	>16
*E. faecium* CTC 492	0.5	1	16	>16
*M. luteus* ATCC 9341	0.063	1	4	8
*S. aureus* ATCC 29213	0.0078	1	4	16
*S. aureus* ATCC 25923	0.0078–0.0156	2	NA^*b*^	NA
*S. aureus* ATCC 43300	0.032	2	16	16
*S. aureus* NCTC 6571	0.032	2	4	16
*S. aureus* ATCC BAA-1720^*c*^	0.0078–0.0156	1	NA	NA
*S. aureus* ATCC 13420^*d*^	0.0078–0.0156	2	NA	NA

^*a*^ The MIC was defined as the concentration where at least one of four parallel cultures exhibited more than 70% reduction in growth compared with untreated control.

^*b*^ MIC was not determined because the condition was not tested.

^*c*^ MRSA252.

^*d*^ Newman.

To test whether MBL-AB01 is likely to be active *in vivo* after injection of a pure compound without applying any stabilizing formulation strategies*,* antibacterial assays against *S. aureus* ATCC43300 (MRSA) and *S. aureus* ATCC29213 (MSSA) were repeated in the presence of 5% and 10% fetal bovine serum (FBS). These assays showed that the MBL-AB01 inhibitory activity was lost in the medium supplemented with FBS. The MBL-AB01 MIC against *S. aureus* ATCC43300 increased by factors of 80 and 300 in the presence of 5% and 10% serum, respectively. The MIC of MBL-AB01 against *S. aureus* ATCC29213 increased by factors of 80 and 750 in the presence of 5% and 10% serum. In contrast, the MIC of streptomycin and vancomycin increased by a maximum of three times under the same conditions (see discussion regarding new strategies to overcome the serum protein binding challenges).

### Structure elucidation of MBL-AB01

Prior to determining the molecular formula of the compound, the number of carbons and nitrogen in the molecule was determined by LC-DAD-QTOF analyses of isotope labeled MBL-AB01 extracts. The observed masses in negative mode for the unlabeled, ^13^C labeled, ^15^N labeled, and ^13^C and ^15^N labeled compounds were m/z 550.0554 Da, m/z 577.1437 Da, m/z 551.0520 Da, and m/z 578.1399 Da, respectively, demonstrating that MBL-AB01 has 27 carbon atoms and 1 nitrogen atom (Fig. S2). The MS spectrum generated with LC-QTOF of the positively charged MBL-AB01 ion, m/z 552.0701, and the corresponding sodium adduct confirmed the mass of MBL-AB01 (Fig. S3). In addition, the isotopic distribution of the MS spectrum shows that the compound is halogenated. The only likely molecular formula for MBL-AB01 satisfying the criteria of 27 carbons, 1 nitrogen, and a halogen was C_27_H_18_ClNO_10_ (1.7 ppm error). The accurate mass and ion formula of purified MBL-AB01 were also verified with ultrahigh resolution Fourier Transform Ion Cyclotron Resonance (FT-ICR), and the measured mass of the negatively charged ion [M-H]^−^ was m/z 550.0549 (−0.4 ppm mass deviation) (Fig. S4; Table S2).

An MS/MS spectrum from positive ionization mode of the candidate mass (Table S3) was submitted (date of submission: 2024-06-18) to GNPS (21), and the MS/MS spectrum of MBL-AB01 did not match any compounds in the spectral library. A screening against the Combined Chemical Dictionary (ChemNetBase) revealed that the molecular formula of the identified compound was identical to that of xantholipin, previously discovered in a *Streptomyces* sp. isolated from a soil sample from China (12). To rule out that MBL-AB01 and xantholipin were the same compound, UV and MS spectra of semi-purified xantholipin (kindly provided by Shanghai Jiao Tong University, China) and MBL-AB01 were compared. In the HPLC chromatograms, MBL-AB01 was observed to elute at 9.1 min, whereas xantholipin eluted earlier at 6.7 min. The UV absorption spectra of the two compounds were also clearly different (Fig. S5). Thus, it was concluded that MBL-AB01 was different from xantholipin.

**Fig 5:**
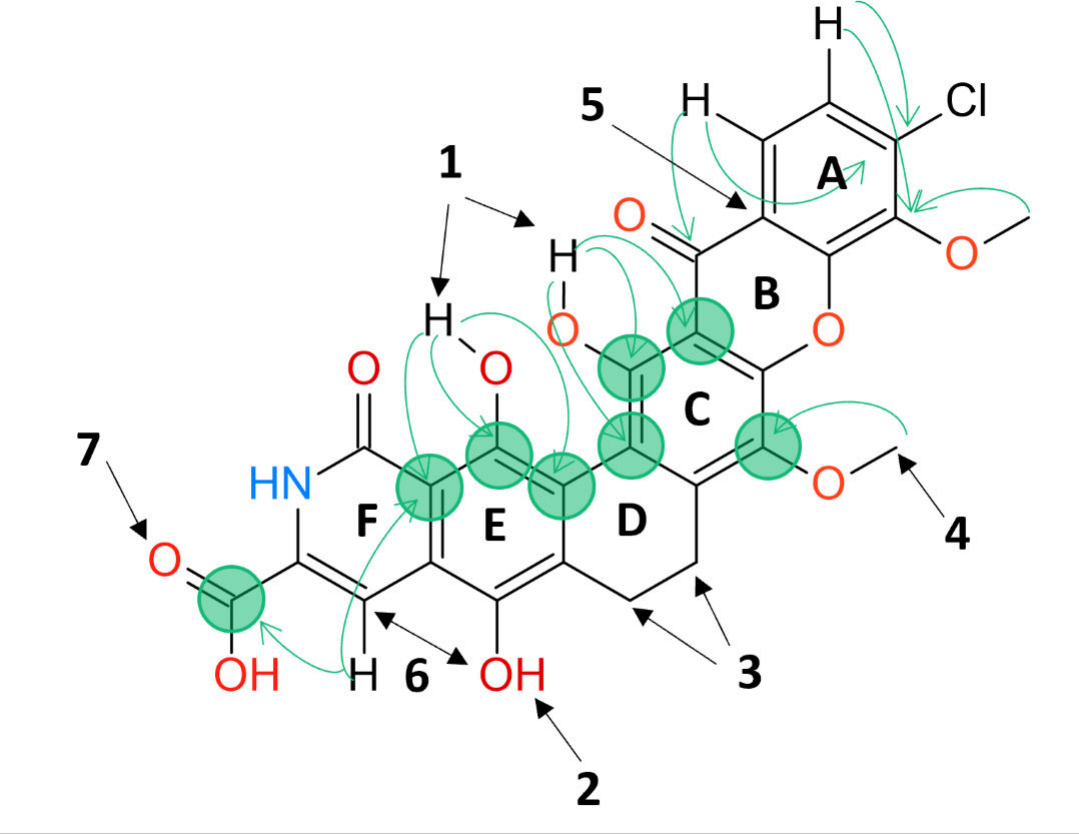
Molecular structure of MBL-AB01 with highlighted key findings (1–7) from the NMR data. Green arrows indicate ^1^H-^13^C long-range correlation found in HMBC NMR spectra. (1) Sharp downfield NMR peaks from OH groups due to hydrogen bonding to nearby carbonyl oxygens. (2) Broad and less downfield NMR peak as compared with the other OH groups due to lack of intramolecular hydrogen bonding. (3) Weakest region with regard to evidence but no contradicting data (the reason for the weak peak intensity may be due to J-couplings and slow ring flips). (4) Additional methoxy group on sp2-carbon verified as compared to Xantholipin. (5) The A and B rings have virtually identical ^1^H and ^13^C chemical shifts to the corresponding part of Xantholipin. (6) A ROE (Rotating-Frame Overhauser Effect) correlation due to dipole-dipole coupling (through-space) between the hydroxyl hydrogen and the nearby aromatic hydrogen is observed. (7) The existence of the carboxyl group was revealed by MS/MS data and subsequently confirmed by the observed ^13^C chemical shifts of the carboxyl carbon. The green circles indicate which ^13^C chemical shifts were identified using ^1^H-^13^C HMBC data.

Fragmentation spectra from FT-ICR analyses of MBL-AB01 indicated the presence of a carboxyl group, most likely a carboxylic acid (neutral loss of CO_2_ from the negatively charged ion m/z 550.0549 to form m/z 506.0644) and hydroxyl group(s) (neutral loss of H_2_O from m/z 506.0644 to form m/z 488.0539, and from m/z 491.0409 to form m/z 473.0304) (Table S2; Fig. S6). Xantholipin also has hydroxyl groups, but the loss of CO_2_ cannot be explained from the structure of xantholipin. The hydrogen-deuterium exchange experiments (Fig. S7; Table S4) suggest the presence of a maximum of five labile hydrogens as the main observed [M-D]^−^ signal m/z 554.0805 corresponds to ion formula [M-D]^−^ with formula C_27_H_13_ClD_4_NO_10_ (mass deviation −1.3 ppm, score 100, msigma 8.3) with four incorporated deuterium. Under oxidative conditions, MBL-AB01 converted to a compound with m/z 550.0540 at positive ionization corresponding to a loss of H_2_. This suggests a conversion of a hydroquinone segment (Ring E, Fig. 5) to the corresponding dione. The observed UV spectrum of the oxidation product exhibited absorption maxima at λmax at 249 nm (58 mAU), 286 nm (55 mAU), and 426 nm (25 mAU), like the dione containing xantholipin (Fig. S5). Acetylation of MBL-AB01 in pyridine and acetic anhydride resulted in m/z 784.100 at positive ionization. This mass corresponds to the sodium adduct of the compound bearing five acetyl groups, indicating that the molecule contains five hydroxyl or amine sites susceptible to acetylation (data not shown).

The key NMR data were obtained from 1D ^13^C (Fig. S8), ^1^H-^13^C HMBC (Fig. S9), ^1^H-^13^C HSQC (Fig. S10), the 2D ^1^H NOESY spectrum (Fig. S11), 2D ^1^H adiabatic ROESY spectrum (Fig. S12), and 1D ^1^H spectrum (Fig. S13). Key data from NMR and MS are summarized in Fig. 5. The structure of MBL-AB01 with atom numbers (Fig. S14), ^1^H and ^13^C chemical shifts from NMR (Table S5), and ^1^H and ^13^C chemical shifts for MBL-AB01 alongside the corresponding values reported for Xantholipin (Table S5) are given in the supplemental material.

^1^H NMR showed 11 proton signals, all integrating for ~1H with the exception of two singlets integrating for 3H at 4.13 and 3.95 ppm which are consistent with –OCH_3_ groups. Three aromatic protons were observed, a pair of doublets (J = 10 Hz) at 7.60 and 7.96 ppm. Both had clear ^1^H-^13^C HSQC correlations and two long-range ^1^H-^13^C HMBC correlations. The final aromatic proton at 7.73/7.46 showed pH sensitivity, a clear ^1^H-^13^C HSQC correlation, and a single long-range ^1^H-^13^C HMBC correlation which overlapped with one observed for the proton at 13.18 ppm. A broad multiplet at 2.4–2.7 ppm overlapped with the residual DMSO-d6 signal and was further confirmed as a CH_2_ group by ^1^H-^13^C HSQC correlation. Two protons at 3.4 ppm were similarly determined, but neither showed long-range ^1^H-^13^C HMBC correlations. Sharp singlets were observed at 13.38, 12.99 ppm, and 9.07 ppm, none of which showed ^1^H-^13^C short-range correlation in HSQC experiments and were thus consistent with three of the five exchangeable protons seen in the hydrogen-deuterium exchange experiments.

The slowly exchangeable protons observed at 12.99 and 13.28 ppm give clear long-range ^1^H-^13^C correlations to three aromatic carbons, each with no overlap. The sharp downfield shift of these protons indicated hydrogen bonding to a second oxygen center. The upfield –OH at 9.07 ppm, by contrast, gave no observable ^1^H-^13^C long-range correlations, but an ROE (Rotating-Frame Overhauser Effect) correlation was observed due to dipole-dipole coupling (through-space) between the two centers. The second –OCH_3_ showed a ^1^H-^13^C long-range HMBC correlation to a single aromatic carbon. A further six carbon centers at 116.7, 133.9, 143.9, 147.6, 166.4, and 173.6 ppm were identified, but these showed no correlations in any of the spectra obtained. A final verification of the proposed structure was performed by comparing the MS/MS fragmentation pattern of MBL-AB01 (Table S3) with the *in silico* fragmentation pattern of the proposed structure. An almost perfect match between the *in silico* and experimental fragmentation patterns provided evidence that the structure was correct (For methods and results, see Fig. S15 in the supplemental material.)

### Gene annotation of the MBL-AB01 cluster

**Fig 6:**
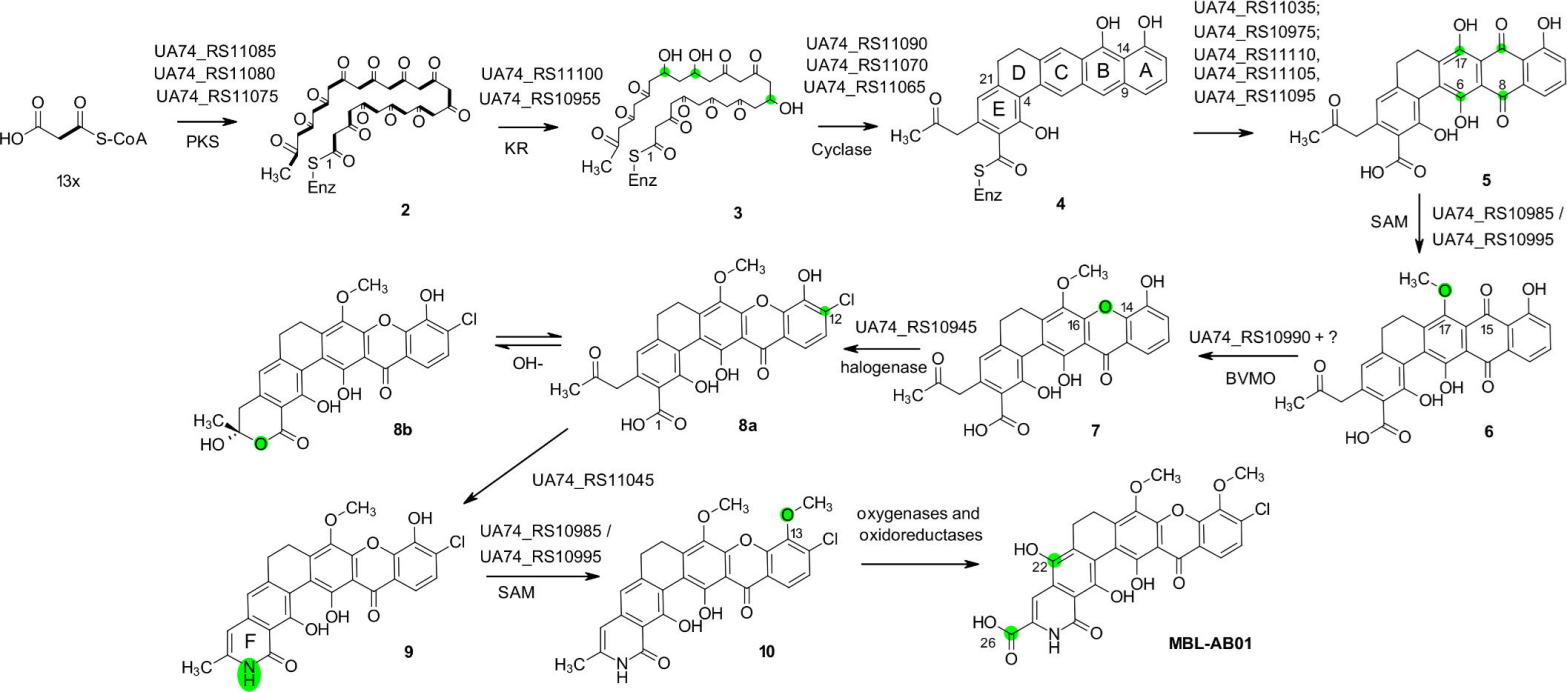
Suggested pathway of MBL-AB01 biosynthesis. The first steps leading to compound 10 coincide with the proposed pathway for xantholipin (22), after which a hydroxyl group is introduced at C22 by an oxygenase, and the C26 methyl group is converted to a carboxylic acid through the combined action of an oxygenase and two oxidoreductases. The identity of the enzymes involved in the last steps is still unknown. Compound numbers for intermediates are the same as in the xantholipin biosynthesis pathway. The most recent modifications that have been introduced are highlighted. Locus_tags for *A. fjordicus* ADI127-7 genes (GenBank accession NZ_CP016076) showing high-sequence similarity to the genes of the xantholipin cluster are indicated (For full description, see Table S4). Minimal PKS: UA74_RS11085 (acyl carrier protein [ACP]), UA74_RS11080 (ketosynthase chain-length factor [CLF]), UA74_RS11075 (beta-ketoacyl-ACP synthase family protein); Keto Reductases (KR): UA74_RS11100 and UA74_RS10955 (SDR family oxidoreductases); cyclases: UA74_RS11090, UA74_RS11070, and UA74_RS11065; the S-adenosyl-L-methionine (SAM)-dependent methyltransferases UA74_RS10985 and UA74_RS10995; the atypical Baeyer-Villiger monooxygenase (BVMO) UA74_RS10990; the halogenase UA74_RS10945; and the asparagine synthetase-like amide synthetase UA74_RS11045. Several monooxygenases and oxidoreductases are found in the cluster. Based on homology to the xan-genes, the generation of five from four possibly involves the monooxygenases UA74_RS10975, UA74_RS11110, UA74_RS11105, and UA74_RS11095 as well as the monooxygenase-like (XanT homolog) UA74_RS11035.

The NMR-based structure elucidation showed that MBL-AB01 is highly similar to two other compounds, xantholipin and lysolipin. Both xantholipin and lysolipin biosynthetic gene clusters (BGCs) have been described before as type II polyketide synthases (PKSs) (22, 23). After genome sequencing and generation of a draft assembly for the strain MP127-IG17, 30 BGCs were predicted by antiSMASH, in which only one cluster of type II PKS was found. Sequence comparisons using BLAST and whole-genome alignment revealed that the MP127-IG17 genome is nearly identical to the previously published *Actinoalloteichus fjordicus* strain ADI127-7 (GenBank: NZ_CP016076) (24), and only a single point mutation was found from raw read mapping. In the published strain, cluster no. 9 is a T2PKS and is indicated to have xanthone as the putative product (24). The antiSMASH analysis indicated that the putative MBL-AB01 cluster shows high similarity to the xantholipin (Minimum Information about a Biosynthetic Gene cluster, MIBiG, ID BGC0000279) (22) and lysolipin BGC (MIBiG ID: BGC0000242) (23) clusters. Assuming the biosynthesis of the three molecules follows a similar mechanism, the MBL-AB01 biosynthetic cluster covers 43 genes (locus_tags UA74_RS10920–UA74_RS11135 in GenBank: NZ_CP016076) (Table S4). Compared to the xantholipin and lysolipin clusters, the MBL-AB01 BGC shows similar function for 37 and 32 of the 43 genes, respectively (Fig. S16; Table S7). The majority of this pathway is conserved with the xantholipin biosynthetic pathway, and genes exhibiting high-sequence similarity were identified for all genes involved in the production of compound 10 (22). After this point, the pathways diverge (Fig. 6).

Malonyl-CoA is possibly synthesized by the combined action of the XanB1-3 homologs UA74_RS11125 (biotin carboxylase), UA74_RS11120 (biotin carboxyl carrier protein), and UA74_RS11115 (carboxyl transferase). Three different S-adenosyl-l-methionine-dependent methyltransferases (SAM-MTases) are identified within the xantholipin biosynthetic cluster, known as XanM1, XanM2, and XanM3. The first two are described as *O*-methyltransferases, whereas XanM3 contains motifs for both *O*- and *C*-methyltransferases (22). Only two candidates were found in the MBL-AB01 cluster, with UA74_RS10995 showing the highest sequence similarity to the *xan*M3 gene, and UA74_RS10985 to the *xan*M2 gene. These two likely transfer a methyl group to the C17 hydroxyl group of compound 5 (Fig. 6) and methylate the C13 hydroxyl groups in the final step before the branching point, producing compound 10. However, it is not known which acts at what step.

To produce MBL-AB01 from compound 10, two additional modifications need to be introduced. A hydroxyl group is added to C22 through the action of an oxygenase, and the C26 methyl group is converted into a carboxylic acid group by the combined actions of multiple enzymes. A monooxygenase first introduces a hydroxyl group, which is then oxidized into an aldehyde and further into the carboxylic acid by one or two oxidoreductases in consecutive steps. It is currently unknown whether the reaction at C22 occurs before or after the reaction at C26. Finally, the transport of the antibiotic (not included in Fig. 6) likely involves UA74_RS11025 (MFS transporter) and UA74_RS11005 (ABC transporter permease).

### Genetic characterization of the MP127-IG17 mutant

Genome sequencing and variant analysis of the classical mutant MP127-IG17(Pl52Br56/E8) revealed four single-base variations and two deletions when compared to the published sequence (24) of the wild-type strain ADI127-7 (Table 3). The 172 bp deletion overlaps a 70 bp region within UA74_RS09135, which encodes a cobyric acid synthase, while the 15 bp deletion is located in UA74_RS33655, which encodes a type I PKS.

**TABLE 3:** Sequence variations found in MP127-IG17(Pl52Br56/E8) compared to the reference strain ADI127-7 (24)

Reference position	Type of mutation	Length (bp)	Reference	Allele	Overlapping locus_tag^*a*^	Changes in coding sequences	Function
105^*b*^	SNV*^c^*	1	A	G	UA74_RS00005	WP_075737760.1: c.A105G	Chromosomal replication initiator protein DnaA
2020291	Deletion	172	GCTGCTCCGGCTCCTCGAGC AGGGCGCCCCGGCCGGGCTG CCGCTGCTGGCGCCGGGGGCATTGCCCTAGCGGCGGTGAC CTCGCCGGCTGTGGCCACCC GATGCGGGCCAGGCGCCGAG CTTCGCTTCAGATCGACGTC GTTGCGGCCGGGGTCGGCTG CCCTGCGCCGCT^*d*^	–*^e^*	UA74_RS09135	WP_318533309.1: c.1521_1590-del GCTGCTCCGGCTCCTCGAG CAGGGCGCCCCGGCCGGGC TGCCGCTGCTGGCGCCGGG GGCATTGCCCTAG; p.L508_P529del^*d*^	Cobyric acid synthase
3749419	Deletion	15	CGACGTCAGGCCGTT	–	UA74_RS33655	WP_075741079.1: c.5557_5571-delAACGGCCTGACGTCG; p.Asn1853_Ser1857del	Type I polyketide synthase
4230442	SNV	1	T	A	UA74_RS18040	WP_075741317.1: c.A2669T; p.Tyr890Phe	Hybrid non-ribosomal peptide synthetase/type I polyketide synthase
4353133	SNV	1	G	C			
6119067	SNV	1	C	T	UA74_RS26110	WP_083683648.1: c.G3250A; p.Val1084Met	S8 family serine peptidase

^*a*^ NCBI GenBank accession NZ_CP016076.

^*b*^ This variation was also seen in the wild-type strain MP127-IG17.

^*c*^ Single-nucleotide variation.

^*d*^ The deletion removes the 3′-end of the gene (70 bp, underlined), including the stop codon, corresponding to 22 C-terminal amino acids (508–529) and 29 new amino acids (PRCVAVVGGRAAASCNRLDQTRSNGTVEI) are added to the C-terminus from the downstream sequence between the deleted sequence and the next stop codon.

^*e*^ No base.

## DISCUSSION

### MBL-AB01 is a novel xanthone compound with a unique structure

**Fig 7:**
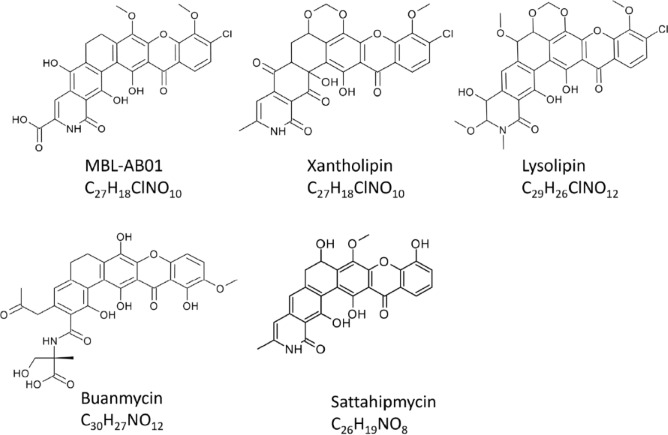
Molecular structures of the five xanthone compounds MBL-AB01, xantholipin, baunmycin, lysolipine, and sattahipmycin.

MBL-AB01 belongs to the xanthone group and shares high similarity with the naturally occurring compounds xantholipin (12, 22), lysolipin (13), and buanmycin (14) (Fig. 7) produced by the *Streptomyces* species, *S. flavogriseus, S. Violaceoniger*, and an uncharacterized *Streptomyces sp.,* respectively. However, to our knowledge, MBL-AB01 is the first bioactive xanthone isolated from a marine *Actinoalloteichus*.

The molecular formula of MBL-AB01 has a very low proton/carbon ratio. The molecular formula of C_27_H_18_ClNO_10_ gave an index of hydrogen deficiency of 19, indicating a high degree of aromaticity and an extensive number of rings in the system, with no evidence of pendant aliphatic groups in the NMR data. The yellow color further supported a highly conjugated system. As most key NMR experiments for structure elucidation (in particular ^1^H 2D NOESY and ^1^H-^13^C HMBC) are proton-detected, this fact complicated the structural assignment by NMR. That is, there are parts of the molecule that are not covered by the accessible through-space or long-range through-bond information from the NMR data, especially concerning non-protonated carbon atoms. Limitations in material availability, solubility, and purity have made the investigations further challenging. Consequently, to successfully resolve the structure of MBL-AB01, the observable chemical shifts and NMR correlations have been combined with several other findings: (i) Chemical shift similarity with the reported compound xantholipin, (ii) the fragmentation pattern from MS/MS analysis, and (iii) the reactivity pattern under specific conditions. The molecular structure of MBL-AB01 is shown in Fig. 5, including a summary of the key structural evidence (see also Tables S5 and S6).

MBL-AB01 and xantholipin share the same molecular formula, and they have a similar substructure. Despite this, the two molecules are significantly different. MBL-AB01 exhibits a core structure with more conjugated double bonds, and the absence of chiral centers in MBL-AB01 suggests a more planar structure than for xantholipin. The flatness, together with higher lipophilicity (logP = 4.8), would explain the observed poor solubility as well as the tendency of dimerization and/or aggregation observed in 2D NOESY spectra.

As MS analysis had shown that MBL-AB01 and xantholipin shared the same molecular formula, an analysis of ^1^H and ^13^C literature data for xantholipin was made with experimental data for MBL-AB01 (12, 22, Table S6). The ^1^H and ^13^C chemical shifts data indicate that xantholipin shares corresponding signals with MBL-AB01 for atom C-1 to C-7 and C-37 and that the A ring and most of the B ring have virtually identical ^1^H and ^13^C chemical shifts to the corresponding part of xantholipin. This is further supported by clear ^1^H-^13^C long-range correlations H-1 → C-5, C-6, H-2 → C-6, C-7 for the aromatic protons, and H-37 → C-5 for the –OCH_3_ protons, and gave a starting point for the structural determination of MBL-AB01 as well as accounting for the location of the Cl observed in the MS analysis. The shifts observed for the rest of MBL-AB01 (rings C to F, including atoms which overlap in rings B and C) are inconsistent with those of xantholipin.

Further fragments were determined by (i) MS analysis indicating a carboxylic acid (CO_2_ loss), (ii) oxidation experiments indicating a quinone ring system, (iii) two phenols on aromatic rings able to hydrogen bond to oxygen centers, but isolated from each other as indicated by ^1^H-^13^C long-range correlations, (iv) an isolated –OCH_3_, and (v) a phenol center without significant hydrogen bonding, but showing an ROE effect to the singlet aromatic proton at 7.73/7.46 ppm (and no ^1^H-^13^C long-range coupling, indicating a more rapid exchange). (vi) Singlet aromatic proton at 7.73/7.46 ppm with an upfield ^13^C shift of 107.2/101.5 ppm (relative to the other proton attached aromatic carbons), indicating a more alkene-like fragment. This ^1^H/^13^C pair was also pH sensitive, without being exchangeable, indicating co-localization with the carboxylic acid fragment. (vii) Two –CH_2_ groups with broad multiplet signals showing only ^1^H-^13^C short-range correlations (HSQC) and no long-range ^1^H-^13^C correlations (HMBC). The multiplet character of these protons indicated –CH_2_–CH_2_– connectivity, most likely in a ring system that can flip slowly.

This information was used to build the structure shown in Fig. 5 starting from the A and B rings, which also satisfies the IHD. Each tentative structure was tested against the evidence obtained, but no alternatives could be found that satisfied all the evidence obtained. For example, exchanging the phenolic –OH on C-11 with the –OCH_3_ at C-14 would result in only one phenol with hydrogen bonding and an up-field shift for the phenol from 13.28 ppm to nearer 9 ppm. Similarly, while swapping –OCH_3_ at C-14 with the phenolic –OH at C-22 would retain the quinone structure but eliminate the ROE effect observed between H-25 and H-30.

In this way, the structure of MBL-AB01 was determined. While several chemical shifts of MBL-AB01 have been marked as “not determined”; in the case of ^1^H, this concerns the rapidly exchanging protons of the amide and the carboxylic acid, and in the case of ^13^C, it concerns quaternary carbons with multi-bond distances to any sharp ^1^H signal. The ^1^D ^13^C data (Fig. S8) confirm the presence of several unassigned ^13^C signals that are consistent with the expected chemical shifts for the suggested structure of MBL-AB01, but atom-specific assignment of these signals is not accessible due to insufficient information from the 2D data. As such, there is no chemical or spectroscopic data that cannot be adequately explained.

### The biosynthesis of MBL-AB01 is likely to follow the same pathway as xantholipin and lysolipin

The BGC of MBL-AB01 was identified, and its 43 genes functionally annotated. Like xantholipin and lysolipin, MBL-AB01 has a scaffold of polycyclic xanthone featuring a cyclic amide (Table S7; Fig. S16). The synthesis of MBL-AB01 diverges from the xantholipin biosynthesis pathway after the introduction of the second *o*-methyl group, and the need for the remaining gene functions has been proposed.

There are several genes coding for monooxygenases in the cluster, where most correspond to enzymes involved in the production of compound 10 (Fig. 6). UA74_RS11000 (homolog to XanO5) has been proposed for C4 hydroxylation. UA74_RS10920 (homolog to XanO2) is a P450 monooxygenase and is suggested to be responsible for methylenedioxy bridge formation. As there is no methylenedioxy bridge in MBL-AB01, UA74_RS10920 could be involved in the hydroxylation of other carbon atoms.

The XanO3 homologs UA74_RS10930 and UA74_RS11135 are both annotated as NAD(P)/FAD-dependent oxidoreductases, but their function in xantholipin biosynthesis remains unassigned, apart from their role as FAD-binding monooxygenases. Other oxidoreductases that have not yet been assigned a specific role include the XanS2 homologs UA74_RS10925 and UA74_RS11055, the XanS1 homolog UA74_RS10965, and the XanZ1 homolog UA74_RS10970. One or more of these enzymes may be involved in the conversion of the C26 methyl group into a carboxylic acid.

### Improving the yield of MBL-AB01

The volumetric yields of active compounds in fermentation processes of *Actinobacteria* are often low, as bacteria in their natural environment do not need to produce large quantities of very potent bioactive compounds. Improving the yields in fermentation processes of poorly studied classes of *Actinobacteria* is often based on trial and error, as little is known about the factors influencing the production of the secondary metabolites in these organisms. Low yields result in cost- and time-consuming production and purification of the bioactive compound, which may lead to a dead end for potential drug candidates. This work demonstrates that the yield of MBL-AB01 was improved by one round of classical UV mutagenesis. The resulting mutations and deletions in the overproducing mutant were not within the BGC of MBL-AB01, and the specific effect of the individual sequence alterations with respect to improved volumetric yields of MBL-AB01 will therefore be speculation. However, the type I PKS affected by the 15 bp deletion was predicted to produce a selvamicin-like compound (25) whose biosynthesis is consuming 17 units of malonyl-CoA, methylmalonyl-CoA, or similar precursors. The biosynthesis of MBL-AB01 requires 13 units of malonyl-CoA (Fig. 6). It is therefore plausible to assume that the 15 bp deletion has reduced the flux of malonyl-CoA into this alternative pathway and thereby increased the supply to the MBL-AB01 pathway. Further mutagenesis trials starting with the candidate selected from the first round are thus a potential route for improving production either by hitting the BGC of MBL-AB01 or competing clusters.

Starting with the PML6 medium originally used to screen the strain collection from which MP127-IG17 originates (17), a new medium (PML6_MOD3) was developed. The highest volumetric yield was obtained in cultivations under high shear forces with pH controlled at 7.5. The extraction using DMSO acidified with trifluoroacetic acid (TFA) significantly improved the yield during purification compared to using pure DMSO. DMSO and TFA are known to improve the solubility of proteins (26).

### MBL-AB01 may have a potential new mode of action

MBL-AB01 demonstrates similar antimicrobial efficacy against both MRSA and methicillin-susceptible *S. aureus* (MSSA) strains, indicating that its activity remains unaffected by the resistance mechanisms that confer the MRSA phenotype. In addition, MBL-AB01 has no structural similarity to the β-lactams to which MRSA strains are resistant nor to the antibiotics currently used to treat MRSA infections, such as vancomycin. Although the exact mode of action of xantholipin remains to be revealed, some information exists. Data indicate that xanthones inhibit efflux pumps, which are important drug resistance mechanisms, possibly in combination with other targets, which vary within the xanthone class of antibiotics (9). Xantholipin originally caught interest due to its anticancer properties, and it was demonstrated to inhibit the heat shock protein HSP47 (12). Xantholipin B, which is produced by a mutant strain of *Streptomyces flocculus* with an inactivated gene encoding aminotransferase, has the same two hydroxyl groups on the E-ring (for ring annotation, see Fig. 5) as MBL-AB01. Whether these hydroxyl groups are important for the activity of MBL-AB01 is unknown. However, it has been demonstrated that the introduction of these two hydroxyl groups in xantholipin B improved its activity against five tumor cell lines but did not enhance antibacterial activity against Gram-negative and Gram-positive bacteria, including the *S. aureus* strain Mu50, which exhibits MRSA and VISA (Vancomycin-Intermediate *S. aureus*) phenotypes (27, 28). This indicates that the xantholipin B mode of action against cancer cell lines differs from that against Gram-positive bacteria.

### Bioactive properties of MBL-AB01 and its potential as a lead antibacterial drug candidate

*In vitro*, MIC of MBL-AB01 shows very high activity against a panel of nine Gram-positive strains, including a vancomycin-resistant *E. faecium* and six MRSA strains. Compared to other related compounds (Fig. 7), MBL-AB01 exhibits equal or higher activity to *S. aureus* strains. Xantholipin inhibits *S. aureus* Mu50 at concentrations of 0.025 µg/mL (28), lysolipin inhibits *S. aureus* Tü 202 at 0.01 µg/mL (29), sattahipmycin inhibits *S. aureus* KB210 at 0.25 µg/mL (15), whereas buanmycin has poor activity against *S. aureus* ATCC 25923 with a MIC of 10.5 µg/mL (14). MBL-AB01 did not exhibit any anticancer effects in human cancer cell lines, nor did it show toxic effects in human non-transformed cell lines under the conditions tested. Interestingly, xantholipin and sattahipmycin demonstrate strong activity against certain cell lines (15, 22) in the presence of serum. Since the growth of human cell lines in culture relies on serum-derived growth factors, we cannot determine whether the absence of toxic effects in cell cultures exposed to MBL-AB01 is due to the presence of serum or whether MBL-AB01 is indeed well tolerated by human cells. Given its exceptionally high *in vitro* potency, it is rational to develop formulations that protect the compound from serum-mediated deactivation. If successful, this will enable *in vivo* efficacy trials and evaluation of toxicity and safety. One strategy, which is under development in our group, is to protect the compound from serum proteins by encapsulation technology and target the formulation toward MRSA (European Innovation Council project number 101046941). Another strategy is to address topical skin infections or prevention of infections in wounds by utilizing nanoparticle carriers that serve as a loading point for the active compound (under development, financed by NordForsk project number 105121). Thus, innovative formulation strategies that aim to overcome challenges encountered *in vivo* are under development.

## MATERIALS AND METHODS

### Strains

MP127-IG17 (17) has been patent deposited under the Budapest Treaty with DSMZ on 7 April 2016, under deposit number DSM 32287. MP127-IG17(Pl52Br56/E8) was developed by UV mutagenesis (this work).

### Cultivation

Seed cultures of MP127-IG17 and the overexpressing candidates derived from this isolate were produced in 500 mL shake flasks containing 100 mL tryptone soya broth medium (Oxoid) supplemented with 0.5 times artificial seawater (17). The cultures were incubated at 30°C for 4 days. For the production cultures, 1% (vol/vol) of inoculum was taken from seed cultures. Initial shake flask productions were performed in 500 mL shake flasks, each containing 125 mL of PML6 medium (17) or PML6_MOD3. The composition of PML6_MOD3 included the following components: 30.0 g/L soluble starch (Sigma), 2.0 g/L peptone (Oxoid), 2.0 g/L yeast extract (Oxoid), 2.5 g/L corn steep liquor (Sigma), and 3.0 g/L CaCO_3_ (Riedel-de Haën). These components were dissolved in water to constitute 75% of the final volume of the medium. After autoclaving, 2× concentrated artificial seawater was added to achieve 25% of the final volume, along with glucose, resulting in the PML6 medium.

Batch fermentations of MP127-17 Pl 52 Br 56 for medium optimization and production of material for purification were performed in 3L bioreactors (Applikon, Delft, Netherlands), inoculated with 1% (vol/vol) from seed cultures. The media tested in the bioreactors were based on PML6_MOD3, but with the following modifications (Table S8): PML6_MOD5: starch replaced with 50 g/L soluble starch from Difco, PML6_MOD6: starch replaced with 50 g/L soluble starch from Difco and supplemented with 7 g/L Na-glutamate (Sigma) and 21 g/L MOPS buffer (Sigma), PML6_MOD7: starch replaced with 50 g/L soluble starch from Difco and supplemented with 2 g/L NH_4_Cl (Sigma) and 21 g/L MOPS (Sigma), PML6_MOD8: starch replaced with 50 g/L soluble starch from Difco, and supplemented with 3.18 g/L NaNO_3_ (Merck) and 21 g/L MOPS buffer (Sigma), PML6_MOD9: starch replaced with 100 g/L soluble starch from Difco, and supplemented with extra 2 g/L yeast extract (Oxoid), 2 g/L peptone (Oxoid), 3 g/L CaCO_3_ (Riedel-de Haën), PML6_MOD10: starch replaced with 50 g/L soluble starch from Difco and supplemented with 2 g/L extra yeast extract (Oxoid), PML6_MOD11: starch replaced with 50 g/L soluble starch from Difco and supplemented with 30 g/L soy flour (Sofarine, BiC, BC’s-Hertogenbosch, Netherlands), PML6_MOD12 starch replaced with 50 g/L soluble starch from Difco, seawater replaced by 0.2 g/L NaCl (VWR), PML6_MOD16: supplemented with 2 g/L yeast extract (Oxoid), 2 g/L peptone (Oxoid) and 2 g/L CaCO_3_ (Riedel-de Haën) and PML6_MOD17: supplemented with additional 2.5 g/L corn steep liquor (Sigma). The pH of the media was adjusted with 2M NaOH to 7.8 from the start. For PML6_MOD9, PML6_MOD10, and PML6_MOD11, PML6_MOD3, PML6_MOD16, and PML6_MOD17, pH was maintained at 7.5 using 2 M NaOH. The other cultivations were run without any pH control. The fill volume was 1.0 L, the temperature was kept at 25°C, and the set point for dissolved oxygen concentration in the bioreactors (controlled by the stirring speed) was initially set at 30%. After 68 hours, the stirring speed was increased to 1,500 rpm (tip speed 350 cm/s) for the bioreactors with PML6_MOD3, PML6_MOD16, and PML6_MOD17. Throughout the fermentation process, the aeration rate (using air) was kept constant at 0.25 vvm (gas volume flow per unit of liquid volume per minute). To minimize foaming, a 10% antifoam (A204, Sigma) suspension in water was used.

The shake flask production of isotope-labeled MBL-AB01 was performed as follows. An unlabeled seed culture was produced in TSB medium supplemented with 2× artificial seawater. A second step seed culture was produced by inoculation of 3% from the unlabeled TSB culture to Silantes *E. coli-*OD2 medium with either ^13^C, ^15^N, or ^13^C+^15^N labeling (Silantes GmbH, Munich, Germany) supplemented with 50% seawater. Then 1% from the second seed step was transferred to production media with the following composition: 537 ml/L *E. coli-*OD2 medium with ^13^C, ^15^N, or ^13^C+^15^N labeling, 0.34 g/L, unlabeled (Sigma) or ^15^N labeled (NH_4_)_2_SO_4_ (Larodan), 0.17 g/L MgSO_4_ × 7H_2_O (Sigma), 2.14 g/L CaCO_3_ (Riedel-de Haën), 0.086 g/L KH_2_PO_4_ (Sigma), 10 g/L unlabeled (Sigma) or ^13^C labeled glucose (Larodan), 1.29 mL/L TMS1 (30). The two seed cultures were incubated for 4 and 6 days, respectively, at 30°C and the production culture was incubated at 25°C for 11 days.

### Extraction and analysis of MBL-AB01

Broth (0.8 g) was transferred to 1.5 mL tubes (Eppendorf), centrifuged, and the supernatant was discarded. The remaining pellet was extracted overnight with 0.8 mL of dimethyl sulfoxide (DMSO) acidified with TFA to a final concentration of 0.1%. The volumetric yield of MBL-AB01 in the fermentation broth was determined using an analytical standard of MBL-AB01 purified with preparative HPLC. To avoid degradation, the standard was prepared by dissolving MBL-AB01 in DMSO to 1.0 mg/mL. The solution was then diluted 10 times with a 50:50 DMSO:KH_2_PO_4_ buffer (5 mM KH_2_PO_4_, with the pH adjusted to 6.0 using KOH. Sodium dithionite was added to 1.1 mg/mL). The solution was flushed with nitrogen prior to storage.

Cell-free extracts were analyzed by an Agilent 1200 Series HPLC system connected to a DAD and either an Agilent 6520 Time of Flight (QTOF) mass spectrometer (Agilent, Santa Clara, CA, USA) or a Bruker Impact II QTOF (Bruker Daltonics, Bremen, Germany). Chromatographic separation was achieved with a Zorbax Bonus-RP column (2.1 × 50 mm, 3.5 µm) using a gradient elution with 0.1% (wt/vol) ammonium acetate in water as mobile phase [A] and acetonitrile as mobile phase [B]. The gradient of mobile phase [B] started at 5% for the first 0.5 min, followed by a linear increase to 95% for the next 25 min. The flow rate was set at 0.3 mL/min, and the column thermostat was maintained at 35°C. UV data were collected from 190 to 600 nm, and the quantitation of MBL-AB01 was performed based on the UV signal at 395 nm.

The Agilent 6520 QTOF mass spectrometer was equipped with an electrospray ionization source, and analyses were performed in both positive and negative ionization modes. The gas temperature was 250°C, the gas flow was 11 L/min, the nebulizer pressure was 40 psi, and the capillary voltage was 3,500 V. Data analysis was performed using MassHunter. The fragmentation spectra were recorded in positive ionization mode using a Bruker Impact II QTOF equipped with an electrospray ionization source. The following parameters were used: Gas temperature: 220°C, gas flow: 10 L/min, nebulizer pressure: 31.9 psi, capillary voltage: 4,500 V, endplate off-set: 500 V, Funnel 1: 400 V, Funnel 2: 600 V, Quadrupole ion energy: 5 eV, Collision Energy: 5 eV, Collision RF: 700–1500 Vpp, Collision energy: 100%–250%, Quadrupole low mass: 300 m/z, Stepping: basic, Transfer time: 20 µS. Collision energy: 30, 40, or 50 V. Data analysis was performed using Data Analysis and Metaboscape (Bruker Daltonics).

### HPLC fractionation and purification of MBL-AB01

Prior to compound discovery, complex cell extracts were fractionated into 24 parts using HPLC as previously described (31). Extracts for HPLC purification of MBL-AB01 were prepared from freeze-dried cell mass. The extraction process for 1 g freeze-dried and homogenized cell mass was as follows. The powder was washed with 50 mL methanol and extracted with 5 mL, then 10 mL DMSO, acidified with TFA to a final concentration of 0.1%. The two DMSO extracts were mixed and then freeze-dried. The resulting dried extract was resuspended in a small amount of DMSO, and any undissolved matter was removed by filtration using a 0.2 µm DMSO-resistant filter. Purification was performed with an Agilent HPLC system equipped with a Zorbax Eclipse XBD-C18 column (9.4 × 250 mm, 5 µm, Agilent) and connected to a DAD and a fraction collector. 20 mM ammonium acetate added 0.4 mL/L of 25% NH_3_ and methanol were used as mobile phases A and B, respectively. The HPLC was run isocratic at 76% [B] for the first 7.5 min, then at 100% [B] from 7.6 to 9.0 min at a flow of 5 mL/min. The active compound eluted at approximately 5.5 min. To avoid degradation of the compound, 50 g/L ammonium acetate buffer (pH = 4.0) was added to each vial in the fraction collector prior to fractionation, giving a final concentration of 0.5 g/L ammonium acetate in the fractions. The fractions were concentrated on an Oasis HLB solid-phase extraction column (Waters, Selangor, Malaysia) that was conditioned with 100% methanol, then 76% methanol with 0.1% 50 g/L ammonium acetate (pH 4.0). After loading the compound onto the column, the column was washed with 1.5 mL 85% methanol (pH = 4) and then with 5 mL 76% methanol (pH = 4). The compound was finally eluted from the column using a methanol-ammonium acetate solution adjusted to pH = 8.0, with a final concentration of 0.05 g/L ammonium acetate. Methanol was removed from the collected eluate by evaporation, and the solid material was resuspended in water and freeze-dried.

### Structure elucidation with high-resolution mass spectrometry and NMR spectroscopy

Mass spectrometry analyses were performed on a Bruker Solarix 12T HRMS (Bruker Daltonics, Bremen, Germany) equipped with an electrospray ionization source. The experiments were performed in both negative and positive ionization modes. The instrument settings were as follows: nebulizer gas pressure 5 bar, dry gas flow rate 10 mL/min and temperature 250°C, capillary voltage 3.5 kV, and an ion accumulation time of 0.15 s. Full mass spectra were recorded from m/z 150–2,000 Da. Fragmentation experiments were performed at collision energies from 5 to 50 eV. Prior to the experiments, mass calibration was performed using a solution of sodium trifluoroacetic acid (NaTFA, 0.1 mg/mL) in acetonitrile:H_2_O (1:1). Data acquisition and analysis were performed using Bruker Data Analysis, including generation and matching against putative ion formulas.

Solution phase hydrogen-deuterium exchange experiments were conducted to elucidate the number of exchangeable hydrogens in the MBL-AB01 molecule. MBL-AB01 was dissolved in deuterated methanol-d4, and high-resolution mass spectrometric analyses were performed after 60 min, 120 min, and after overnight storage. The mass spectra were recorded and compared to those of MBL-AB01 dissolved in non-deuterated methanol at the same concentration to elucidate the maximum number of exchangeable hydrogens. Back exchange experiments for verifying the reversible exchange of deuterium to hydrogen were also performed by diluting fully exchanged (after overnight exchange in methanol-d4) MBL-AB01 in methanol and comparing the results to those of MBL-AB01 dissolved in methanol. Acetylation of MBL-AB01 was performed by dissolving 1.2 mg MBL-AB01 in 0.5 mL pyridine, followed by the addition of 0.4 mL acetic acid anhydride. After stirring for 18 hours, the mixture was concentrated, co-evaporated from toluene three times, and analyzed by LC-MS.

An 800 MHz Bruker Avance spectrometer equipped with a 5 mm CPTXO ^13^C/^15^N-^1^H/D Z-GRD (Z140404/0001) probe was used to record 1D ^1^H, 1D ^13^C, 2D ^13^C HSQC, and ^13^C HMBC spectra. A 500 MHz Varian Inova spectrometer equipped with a 5 mm ^1^H/^13^C/^15^N triple resonance probe was used for all other performed NMR experiments, including standard versions of 1D ^1^H, 2D ^1^H gradient-COSY, 2D ^1^H TOCSY (mixing time = 60 ms), ^1^H-^13^C HSQC (optimized for selected regions), ^1^H-^13^C HMBC (optimized for 8 Hz), 2D ^1^H NOESY (mixing time = 400 ms), and 2D ^1^H adiabatic ROESY (mixing time = 200 ms). All spectra were recorded at 25°C. All NMR spectra were processed and analyzed using MestreNova 9.0.0.

A 0.7 mg sample of MBL-AB01 was dissolved in 180 µL of DMSO-d_6_, transferred to a 3 mm NMR tube, and flushed with nitrogen gas before capping the NMR tube. The NMR sample was analyzed at 800 MHz immediately after preparation, using 1D ^1^H spectra to assess sample purity and monitor stability over time. The sample was kept at 0°C prior to continued data collection at 500 MHz.

### Whole-genome sequencing and bioinformatics

Total DNA was extracted using the MasterPure Gram-Positive DNA Purification Kit (Biosearch Technologies, UK). The concentration of the extracted DNA was measured fluorometrically using the Qubit dsDNA Quantification, Broad Range kit (Thermo Fisher Scientific). DNA integrity and size distribution were evaluated by agarose gel electrophoresis. DNA sequencing libraries were generated using the Nextera XT DNA Library Preparation Kit (Illumina) in combination with a relevant indexing kit and sequenced in the 2 × 300 bp PE mode on a MiSeq (Illumina, California, USA).

Sequencing reads were demultiplexed in MiSeq control software (Illumina). All subsequent bioinformatics operations were then performed in CLC Genomics Workbench v.24 (Qiagen, Aarhus, Denmark) (CLCGWB) with standard settings, unless otherwise specified. Raw reads were trimmed using the Trim Reads 3.0 tool, and draft assemblies were generated using the *De Novo* assembly tool 1.5. The Basic Local Alignment Search Tool (BLAST) 1.2 running the BLASTN version 2.16.1 + was used to identify hits in the nucleotide collection (nr/nt) database from NCBI. The most relevant hits were downloaded and used for whole-genome alignment analysis using the Create Whole Genome Alignment tool (version 1.0), followed by the Create Average Nucleotide Identity Comparison tool (version 1.0), and as templates for read mapping. Trimmed raw reads were mapped against reference genomes using the Map Reads to Reference tool (version 1.9) in the “Create stand-alone read mappings” output mode. Sequence variants were called using the Basic Variant Detection tool (version 2.6), while larger deletions were detected by manual inspection of the regions with no coverage found in the read tracks.

To enhance the completeness of genome assembly, long-read sequence data were generated by nanopore sequencing on a MinION sequencer (Oxford Nanopore Technologies, UK). DNA libraries were generated using the 1D Native Barcoding Genomic DNA Kit (EXP-NBD103 and SQK-LSK108) and sequenced on a FLO-MIN106 flow cell for 39 hours, controlled by the MinKNOW software. The raw reads (fast5-format) were base-called and demultiplexed using Albacore version 2.1.3. Adapter sequence removal and barcode verification were performed using Porechop (https://github.com/rrwick/Porechop) version 0.2.3. Quality-trimmed Illumina sequencing reads were exported from CLCGWB. A *de novo* assembly was created by Unicycler version 0.4.4 (32), using both the short Illumina reads and the long MinION reads. Unicycler was accessed through the Galaxy server (33), hosted by the University of Tromsø (https://usegalaxy.no/). The Unicycler hybrid assembly was further refined through multiple rounds in CLCGWB. Trimmed Illumina reads were mapped against the assembly, sequence variants called, and the consensus sequence extracted using the Extract Consensus Sequence tool (version 1.3). This consensus sequence served as a template for an additional round of read mapping until no more high-frequency sequence variants were detected or until the number of variants no longer decreased.

### Gene cluster annotation

Genome assemblies were analyzed for potential BGCs using a local installation of the software antiSMASH (antibiotics & Secondary Metabolite Analysis SHell) bacterial version 4.2.0dev. The software was downloaded from the website (https://antismash.secondarymetabolites.org/#!/download), installed, and run on the High-Performance-Computing (HPC) platform at SINTEF. Information for reference genes from the xantholipin or lysolipin BGCs was extracted from the antiSMASH results. Genes that did not match any reference genes from the xantholipin or lysolipin reference clusters were annotated using a manual BLAST search against the nucleotide collection (nr/nt) database of NCBI. The alignment and visualization of the clusters (Fig. S16) was carried out using clinker & clustermap.js (34).

### *In vitro* antibacterial and cytotoxicity assays

An antibacterial assay, conducted to identify the active HPLC fractions, was performed as described previously (31) with *M. luteus* ATCC 9341 and *E. faecium* CCUG 37832 as indicator organisms. The MICs of HPLC-purified MBL-AB01 were determined by standardized microdilution tests, as described previously (35, 36). The MBL-AB01 was tested against the following strains: *E. faecium* CCUG 37832, *E. faecium* CTC 492, *M. luteus* ATCC 9341, *S. aureus* ATCC 29213 (MSSA), *S. aureus* ATCC 25923 (MSSA), *S. aureus* NCTC 6571 (MSSA), *S. aureus* ATCC 43300 (MRSA), *S. aureus* ATCC BAA-1720 (MRSA, MRSA252), and *S. aureus* ATCC 13420 (MSSA, Newman). A Beckman Coulter Biomek NXP and SCARA robotic system was used to disperse 30 µL of LAB114 Mueller Hinton Broth (LAB M Ltd, Lancashire, UK) which contained a twofold dilution series of MBL-AB01 and control antibiotics (vancomycin, gentamicin, and streptomycin), into clear, flat, and sterile polystyrene 384-microwell plates (Nunc 242757). For the protein-binding assays, the Mueller-Hinton broth was supplemented with either 5% or 10% FBS (Sigma F7524). Subsequently, a Tecan Freedom EVO-2 200 robot system, equipped with an MCA384 well pipetting tool, was used to add 7.5 µL of bacterial inoculum containing 5 × 10^5^ CFU/mL to the assay plates. The plates were shaken at 2,000 rpm for 20 seconds on a Quantifoil Instruments GmbH BioShake 3000 Elm BioShake and incubated for 19 h at 34°C. All tests were performed in quadruplicate, and MIC was determined as the lowest concentration where at least one of four replicate cultures showed more than 70% reduction in growth compared with untreated control.

*In vitro* cytotoxicity was evaluated using human cancer cell lines SF-295, OVCAR-3, COLO 205, DU-145, and SW-620 as described previously (37–39), along with the non-cancer cell line IMR90 human lung fibroblast (ATCC CCL-1 86). The IMR90 cells were cultured in DMEM-low glucose (Sigma) supplemented with 10% FBS (Sigma), 2 mM L-glutamine, 1% MEM NEAA (Sigma), 1 mM sodium pyruvate, 10 mM HEPES, and 100 U/mL penicillin-streptomycin. The cancer cell lines were grown in RPMI 1640 (Thermo Fisher Scientific) supplemented with 10% FBS, 2 mM L-glutamine, 10 mM HEPES, and 100 U/mL penicillin-streptomycin. Depending on their confluency, the cells were sub-cultured two or three times a week at ratios ranging from 1:2 to 1:8. On the day before exposing the cells to the compounds, 30 µL of cell suspension containing 1.2 × 10^5^ cells per mL was seeded into 384-well plates (Assay Plate, 3712, Corning, New York, USA) using a Tecan EVO robotic workstation (Tecan, Männedorf, Switzerland) equipped with MCA384 pipetting unit and disposable tips (Tecan MCA 125 µL, Cat No. 300-5-1-808). The cell suspension was transferred to the microplates from a 300 mL stirred tank (Reservoir flat base 10723363), where it was stirred using sterile magnetic stirring bars (15 × 4.5 mm VWR 442-4522) at 350 rpm on the Tecan EVO. After seeding, the microplates containing the cell suspension were shaken at 1,600 rpm with an amplitude of 2.5 mm (Bioshake, Tecan) for 20 seconds. The microplates were then incubated at 37°C with a 5% CO_2_ atmosphere. On the day of exposure, serial dilutions of the compounds were made in DMSO. These dilutions were further diluted in a cell culture medium and transferred to the assay wells, giving a total DMSO concentration of 0.6% in each well. After the exposure period, the plates were further incubated at 37°C with a 5% CO_2_ atmosphere for 24 hours. The viability of the cells after incubation was measured using the Promega CellTiter-GLO 2.0 luminescent cell viability assay (Promega, Wisconsin, USA).

### Classical mutagenesis with UV radiation

Spores of MP127-IG17 were produced on ISP2 agar supplemented with 0.5 × artificial seawater and harvested into sterile ion-free water containing 0.9% NaCl and 0.1% Tween 80. The spore suspension was diluted twofold with 0.5× artificial seawater to OD_600_ = 0.89. Then, 2.5 mL of the diluted spore suspension was added to each well of a six-well microtiter plate (Sarstedt 83.1839.500) and placed on an Infors AG laboratory shaker. The samples were exposed to UV light 254 nm for varying durations ranging from 0 to 420 seconds, using a UV lamp (UVP, Model UVGL-58, 6 watts, Analytic Jena, California, USA) positioned 75 mm from the meniscus of the spore suspension, while being shaken at 180 rpm with a 15 mm amplitude. After exposure, the 2.5 mL spore suspension was transferred to a light-blocking 50 mL tube containing 2.5 mL LB medium with 0.5× artificial seawater. The tubes were incubated at 30°C with shaking at 200 rpm and a 25 mm amplitude for 50 min. Following this incubation, glycerol was added to each well to achieve a final concentration of 15% (vol/vol), and samples were stored at −80°C. Prior to freezing, a dilution series of the samples was plated on LA agar containing artificial seawater to determine the killing rate at different exposure times. Subsequently, the stored samples were thawed and plated on LA agar with artificial seawater. A total of 10,752 colonies were picked robotically using a Genetix QPixII robot (Labexchange, Burladingen, Germany) and transferred to 96-well microtiter plates (655261, Greiner Bio-One, Kremsmünster, Austria), each containing 125 µL of 0.5× LB medium with 50% artificial seawater, along with two 3 mm glass beads per well. These colonies were cultivated for 9 days at 30°C with shaking at 800 rpm. After cultivation, 5 µL aliquots of the cultures were transferred to new 96-well microtiter plates (Greiner 650261) containing 125 µL of 0. 3× PML6_MOD3 with 50% artificial seawater. Glycerol was then added to each well containing LB culture, and the plates were stored at −80°C. The PML6_MOD3 cultures were incubated for 8 days at 25°C with shaking at 800 rpm. On day 8, the cultures were pelleted, and the supernatant was discarded. The pellets were extracted overnight with DMSO that had been acidified with TFA to a final concentration of 0.1%. The DMSO extracts prepared from the cell pellets were tested for activity against *E. faecium* CTC492 in a robotic antimicrobial assay. The bioassay was performed according to the MIC protocol described earlier, but only two dilutions of the DMSO extracts from each mutant were used instead of a complete dilution series.

### Conclusion

The novel compound MBL-AB01 is a promising candidate for the treatment of multi-resistant infections. The compound belongs to the group of natural xanthones. The structure of the compound has been determined, and a gene cluster that encodes the enzymes involved in its biosynthesis has been identified and annotated. This compound demonstrates potential effectiveness against Gram-positive bacteria and may be a promising candidate for treating infections caused by multidrug-resistant bacteria. Additionally, a method has been developed to produce the MBL-AB01 compound in sufficient quantities for further formulation and testing.

## Data Availability

Raw sequencing reads have been deposited to the Sequence Read Archive (SRA) under Bioproject PRJNA1194358.

## SUPPLEMENTAL MATERIAL

Supplemental material aem.01346-25-s0001.pdf Figures S1 to S16; Tables S1 to S8.

ASM does not own the copyrights to Supplemental Material that may be linked to, or accessed through, an article. The authors have granted ASM a non-exclusive, world-wide license to publish the Supplemental Material files. Please contact the corresponding author directly for reuse.

## References

[1] Murray CJL, Ikuta KS, Sharara F, Swetschinski L, Robles Aguilar G, Gray A, Han C, Bisignano C, Rao P, Wool E, et al.. 2022. Global burden of bacterial antimicrobial resistance in 2019: a systematic analysis. Lancet 399:629–655. doi:10.1016/S0140-6736(21)02724-0 35065702 PMC8841637

[2] World Health Organization. 2019. 2019 antibacterial agents in clinical development: an analysis of the antibacterial clinical development pipeline. World Health Organization, Geneva. Available from: https://apps.who.int/iris/handle/10665/330420. Retrieved 8 Sep 2023.

[3] Poon H, Chang MH, Fung HB. 2012. Ceftaroline fosamil: a cephalosporin with activity against methicillin-resistant Staphylococcus aureus. Clin Ther 34:743–765. doi:10.1016/j.clinthera.2012.02.025 22444785

[4] Noel GJ. 2007. Clinical profile of ceftobiprole, a novel beta-lactam antibiotic. Clin Microbiol Infect 13 Suppl 2:25–29. doi:10.1111/j.1469-0691.2007.01725.x 17488373

[5] Gajdács M. 2019. The continuing threat of methicillin-resistant Staphylococcus aureus. Antibiotics (Basel) 8:52. doi:10.3390/antibiotics8020052 31052511 PMC6627156

[6] Kawasuji H, Nagaoka K, Tsuji Y, Kimoto K, Takegoshi Y, Kaneda M, Murai Y, Karaushi H, Mitsutake K, Yamamoto Y. 2023. Effectiveness and safety of linezolid versus vancomycin, teicoplanin, or daptomycin against methicillin-resistant Staphylococcus aureus bacteremia: a systematic review and meta-analysis. Antibiotics (Basel) 12:697. doi:10.3390/antibiotics12040697 37107059 PMC10135165

[7] Vestergaard M, Frees D, Ingmer H. 2019. Antibiotic resistance and the MRSA problem. Microbiol Spectr 7:7. doi:10.1128/microbiolspec.gpp3-0057-2018 PMC11590431 30900543

[8] Hug JJ, Bader CD, Remškar M, Cirnski K, Müller R. 2018. Concepts and methods to access novel antibiotics from Actinomycetes. Antibiotics (Basel) 7:44. doi:10.3390/antibiotics7020044 29789481 PMC6022970

[9] Durães F, Resende D, Palmeira A, Szemerédi N, Pinto MMM, Spengler G, Sousa E. 2021. Xanthones active against multidrug resistance and virulence mechanisms of bacteria. Antibiotics (Basel) 10:600. doi:10.3390/antibiotics10050600 34069329 PMC8158687

[10] Liang M, Ge X, Xua H, Ma K, Zhang W, Zan Y, Efferth T, Xue Z, Hua X. 2022. Phytochemicals with activity against methicillin-resistant Staphylococcus aureus. Phytomedicine 100:154073. doi:10.1016/j.phymed.2022.154073 35397285

[11] Soares JX, Loureiro DRP, Dias AL, Reis S, Pinto MMM, Afonso CMM. 2022. Bioactive marine xanthones: a review. Mar Drugs 20:58. doi:10.3390/md20010058 35049913 PMC8778107

[12] Terui Y, Yiwen C, Jun-ying L, Ando T, Yamamoto H, Kawamura Y, Tomishima Y, Uchida S, Okazaki T, Munetomo E, Seki T, Yamamoto K, Murakami S, Kawashima A. 2003. Xantholipin, a novel inhibitor of HSP47 gene expression produced by Streptomyces sp. Tetrahedron Lett 44:5427–5430. doi:10.1016/S0040-4039(03)01318-2

[13] Drautz H, Keller-Schierlein W, Zähner H. 1975. Metabolic products of microorganisms, 149. Lysolipin I, a new antibiotic from Streptomyces violaceoniger (author’s transl). Arch Microbiol 106:175–190. doi:10.1007/BF00446521 814871

[14] Moon K, Chung B, Shin Y, Rheingold AL, Moore CE, Park SJ, Park S, Lee SK, Oh K-B, Shin J, Oh D-C. 2015. Pentacyclic antibiotics from a tidal mud flat-derived actinomycete. J Nat Prod 78:524–529. doi:10.1021/np500736b 25495422

[15] Leetanasaksakul K, Koomsiri W, Suga T, Matsuo H, Hokari R, Wattana-Amorn P, Takahashi YK, Shiomi K, Nakashima T, Inahashi Y, Thamchaipenet A. 2022. Sattahipmycin, a hexacyclic xanthone produced by a marine-derived Streptomyces. J Nat Prod 85:1211–1217. doi:10.1021/acs.jnatprod.1c00870 35512262

[16] Karthikeyan A, Joseph A, Nair BG. 2022. Promising bioactive compounds from the marine environment and their potential effects on various diseases. J Genet Eng Biotechnol 20:14. doi:10.1186/s43141-021-00290-4 35080679 PMC8790952

[17] Engelhardt K, Degnes KF, Kemmler M, Bredholt H, Fjaervik E, Klinkenberg G, Sletta H, Ellingsen TE, Zotchev SB. 2010. Production of a new thiopeptide antibiotic, TP-1161, by a marine Nocardiopsis species. Appl Environ Microbiol 76:4969–4976. doi:10.1128/AEM.00741-10 20562278 PMC2916467

[18] Zhang H, Zheng W, Huang J, Luo H, Jin Y, Zhang W, Liu Z, Huang Y. 2006. Actinoalloteichus hymeniacidonis sp. nov., an actinomycete isolated from the marine sponge Hymeniacidon perleve. Int J Syst Evol Microbiol 56:2309–2312. doi:10.1099/ijs.0.64217-0 17012552

[19] Ding L, Zhang S-D, Haidar AK, Bajimaya M, Guo Y, Larsen TO, Gram L. 2021. Polycyclic tetramate macrolactams-a group of natural bioactive metallophores. Front Chem 9:772858. doi:10.3389/fchem.2021.772858 34869220 PMC8632820

[20] Schulz S, Sletta H, Fløgstad Degnes K, Krysenko S, Williams A, Olsen SM, Vernstad K, Mitulski A, Wohlleben W. 2023. Optimization of FK-506 production in Streptomyces tsukubaensis by modulation of crp-mediated regulation. Appl Microbiol Biotechnol 107:2871–2886. doi:10.1007/s00253-023-12473-9 36949330 PMC10033298

[21] Wang M, Carver JJ, Phelan VV, Sanchez LM, Garg N, Peng Y, Nguyen DD, Watrous J, Kapono CA, Luzzatto-Knaan T, et al.. 2016. Sharing and community curation of mass spectrometry data with global natural products social molecular networking. Nat Biotechnol 34:828–837. doi:10.1038/nbt.3597 27504778 PMC5321674

[22] Zhang W, Wang L, Kong L, Wang T, Chu Y, Deng Z, You D. 2012. Unveiling the post-PKS redox tailoring steps in biosynthesis of the type II polyketide antitumor antibiotic xantholipin. Chem Biol 19:422–432. doi:10.1016/j.chembiol.2012.01.016 22444597

[23] Lopez P, Hornung A, Welzel K, Unsin C, Wohlleben W, Weber T, Pelzer S. 2010. Isolation of the lysolipin gene cluster of Streptomyces tendae Tü 4042. Gene 461:5–14. doi:10.1016/j.gene.2010.03.016 20399259

[24] Nouioui I, Rückert C, Willemse J, van Wezel GP, Klenk H-P, Busche T, Kalinowski J, Bredholt H, Zotchev SB. 2017. Actinoalloteichus fjordicus sp. nov. isolated from marine sponges: phenotypic, chemotaxonomic and genomic characterisation. Antonie Van Leeuwenhoek 110:1705–1717. doi:10.1007/s10482-017-0920-9 28770445 PMC5676828

[25] Van Arnam EB, Ruzzini AC, Sit CS, Horn H, Pinto-Tomás AA, Currie CR, Clardy J. 2016. Selvamicin, an atypical antifungal polyene from two alternative genomic contexts. Proc Natl Acad Sci USA 113:12940–12945. doi:10.1073/pnas.1613285113 27803316 PMC5135293

[26] Houen G, Bechgaard K, Bechgaard K, Songstad J, Leskelä M, Polamo M, Homsi MN, Kuske FKH, Haugg M, Trabesinger-Rüf N, Weinhold EG. 1996. The solubility of proteins in organic solvents. Acta Chem Scand 50:68–70. doi:10.3891/acta.chem.scand.50-0068

[27] Hiramatsu K, Aritaka N, Hanaki H, Kawasaki S, Hosoda Y, Hori S, Fukuchi Y, Kobayashi I. 1997. Dissemination in Japanese hospitals of strains of Staphylococcus aureus heterogeneously resistant to vancomycin. Lancet 350:1670–1673. doi:10.1016/S0140-6736(97)07324-8 9400512

[28] Wu S, Huang T, Xie D, Wo J, Wang X, Deng Z, Lin S. 2017. Xantholipin B produced by the stnR inactivation mutant Streptomyces flocculus CGMCC 4.1223 WJN-1. J Antibiot 70:90–95. doi:10.1038/ja.2016.60 27328868

[29] Drautz H, Keller-Schierlein W, Zähner H. 1975. Stoffwechselprodukte von mikroorganismen: 149. Mitteilung. lysolipin i, ein neues antibioticum aus Streptomyces violaceoniger. Arch Microbiol 106:175–190. doi:10.1007/bf00446521 814871

[30] Sekurova O, Sletta H, Ellingsen TE, Valla S, Zotchev S. 1999. Molecular cloning and analysis of a pleiotropic regulatory gene locus from the nystatin producer Streptomyces noursei ATCC11455. FEMS Microbiol Lett 177:297–304. doi:10.1111/j.1574-6968.1999.tb13746.x 10474196

[31] Králová S, Sandoval-Powers M, Fawwal DV, Degnes KF, Lewin AS, Klinkenberg G, Nguyen G-S, Liles MR, Wentzel A. 2021. Streptomyces tardus sp. nov.: a slow-growing actinobacterium producing candicidin, isolated from sediments of the Trondheim fjord. Front Microbiol 12:714233. doi:10.3389/fmicb.2021.714233 34421874 PMC8371330

[32] Wick RR, Judd LM, Gorrie CL, Holt KE. 2017. Unicycler: resolving bacterial genome assemblies from short and long sequencing reads. PLoS Comput Biol 13:e1005595. doi:10.1371/journal.pcbi.1005595 28594827 PMC5481147

[33] Afgan E, Baker D, van den Beek M, Blankenberg D, Bouvier D, Čech M, Chilton J, Clements D, Coraor N, Eberhard C, Grüning B, Guerler A, Hillman-Jackson J, Von Kuster G, Rasche E, Soranzo N, Turaga N, Taylor J, Nekrutenko A, Goecks J. 2016. The galaxy platform for accessible, reproducible and collaborative biomedical analyses: 2016 update. Nucleic Acids Res 44:W3–W10. doi:10.1093/nar/gkw343 27137889 PMC4987906

[34] Gilchrist CLM, Chooi Y-H. 2021. Clinker & clustermap.js: automatic generation of gene cluster comparison figures. Bioinformatics 37:2473–2475. doi:10.1093/bioinformatics/btab007 33459763

[35] Khan S, Tøndervik A, Sletta H, Klinkenberg G, Emanuel C, Onsøyen E, Myrvold R, Howe RA, Walsh TR, Hill KE, Thomas DW. 2012. Overcoming drug resistance with alginate oligosaccharides able to potentiate the action of selected antibiotics. Antimicrob Agents Chemother 56:5134–5141. doi:10.1128/AAC.00525-12 22825116 PMC3457396

[36] Tøndervik A, Sletta H, Klinkenberg G, Emanuel C, Powell LC, Pritchard MF, Khan S, Craine KM, Onsøyen E, Rye PD, Wright C, Thomas DW, Hill KE. 2014. Alginate oligosaccharides inhibit fungal cell growth and potentiate the activity of antifungals against Candida and Aspergillus spp. PLoS One 9:e112518. doi:10.1371/journal.pone.0112518 25409186 PMC4237368

[37] Sulheim E, Iversen T-G, To Nakstad V, Klinkenberg G, Sletta H, Schmid R, Hatletveit AR, Wågbø AM, Sundan A, Skotland T, Sandvig K, Mørch Ý. 2017. Cytotoxicity of poly(alkyl cyanoacrylate) nanoparticles. Int J Mol Sci 18:2454. doi:10.3390/ijms18112454 29156588 PMC5713421

[38] Flobak Å, Niederdorfer B, Nakstad VT, Thommesen L, Klinkenberg G, Lægreid A. 2019. A high-throughput drug combination screen of targeted small molecule inhibitors in cancer cell lines. Sci Data 6:237. doi:10.1038/s41597-019-0255-7 31664030 PMC6820772

[39] Folkesson E, Niederdorfer B, Nakstad VT, Thommesen L, Klinkenberg G, Lægreid A, Flobak Å. 2020. High-throughput screening reveals higher synergistic effect of MEK inhibitor combinations in colon cancer spheroids. Sci Rep 10:11574. doi:10.1038/s41598-020-68441-0 32665693 PMC7360566

