# Supplementary material for: Discovery of MBL-AB01: a novel antibacterial xanthone antibiotic with high activity against methicillin-resistant Staphylococcus aureus
Source: Appl Environ Microbiol. 2025 Dec 8;92(1):e01346-25. doi: 10.1128/aem.01346-25 (PMC12838262; doi:10.1128/aem.01346-25)
Supplement: Supplemental material — Figures S1 to S16; Tables S1 to S8. [file aem.01346-25-s0001.pdf]

Supplementary information to manuscript:

Degnes et al. " Discovery of MBL-AB01: A novel antibacterial xanthone antibiotic with high activity against methicillin-resistant *Staphylococcus aureus*"

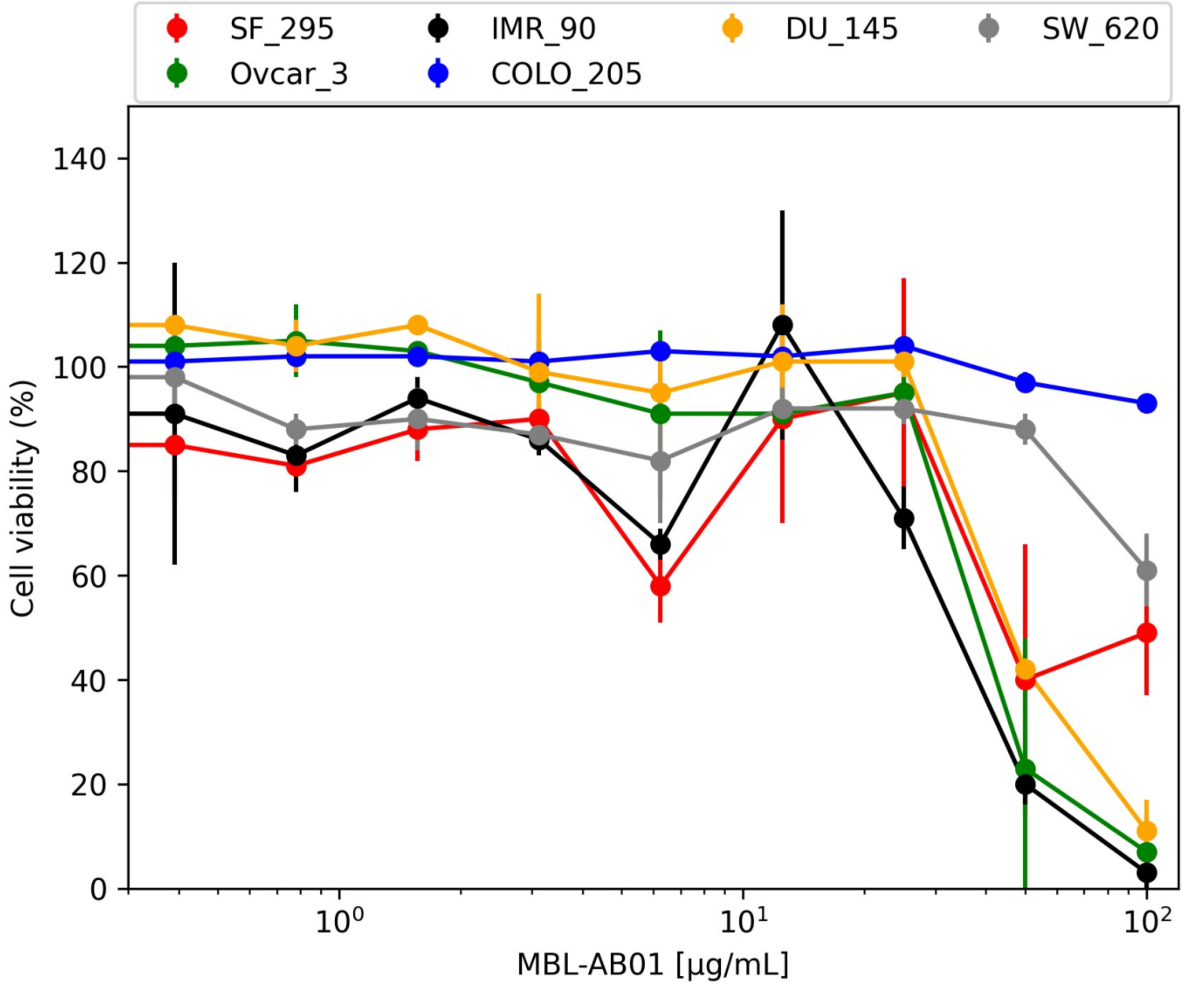

Fig. S1 Cell survival of one non-transformed cell line (IMR-90) and five cancer cell lines after exposure to MBL-AB01. No toxic effect was observed at 25 µg/mL. The cell viability of exposed cells is given as percent the cell viability of non-exposed cells. The assay was performed with two replicates, and the error bars represent the highest and lowest measured values, given as percent of the cell viability of non-exposed cells, at each data point.

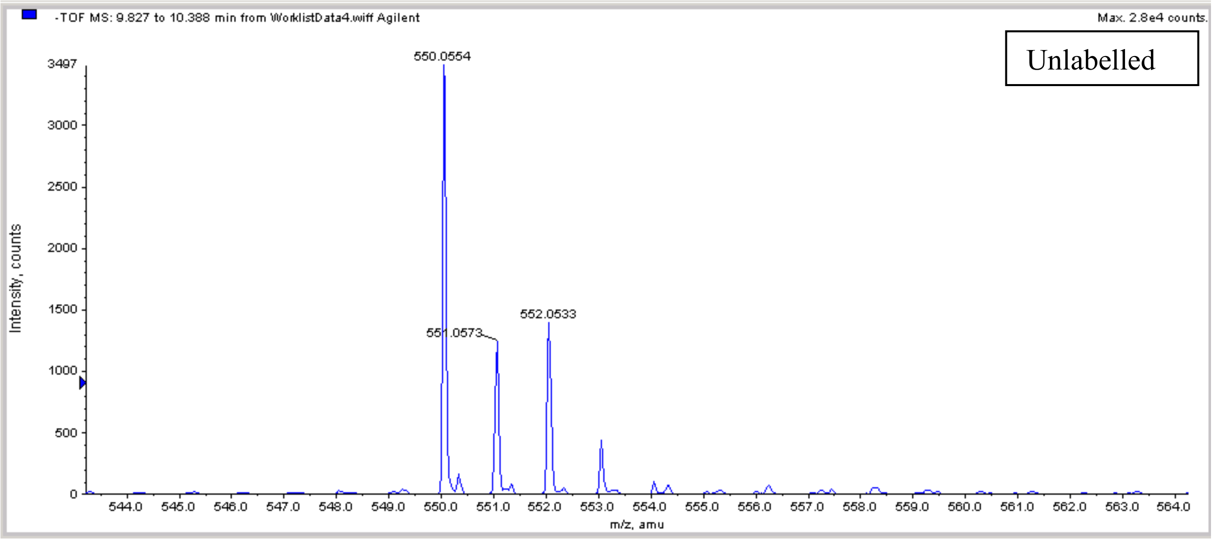
-TOF MS: 9.827 to 10.388 min from WorklistData4.wiff Agilent
Max. 2.8e4 counts.
Unlabelled
550.0554
551.0573
552.0533
Intensity, counts
3497
3000
2500
2000
1500
1000
500
0
544.0 545.0 546.0 547.0 548.0 549.0 550.0 551.0 552.0 553.0 554.0 555.0 556.0 557.0 558.0 559.0 560.0 561.0 562.0 563.0 564.0
m/z, amu

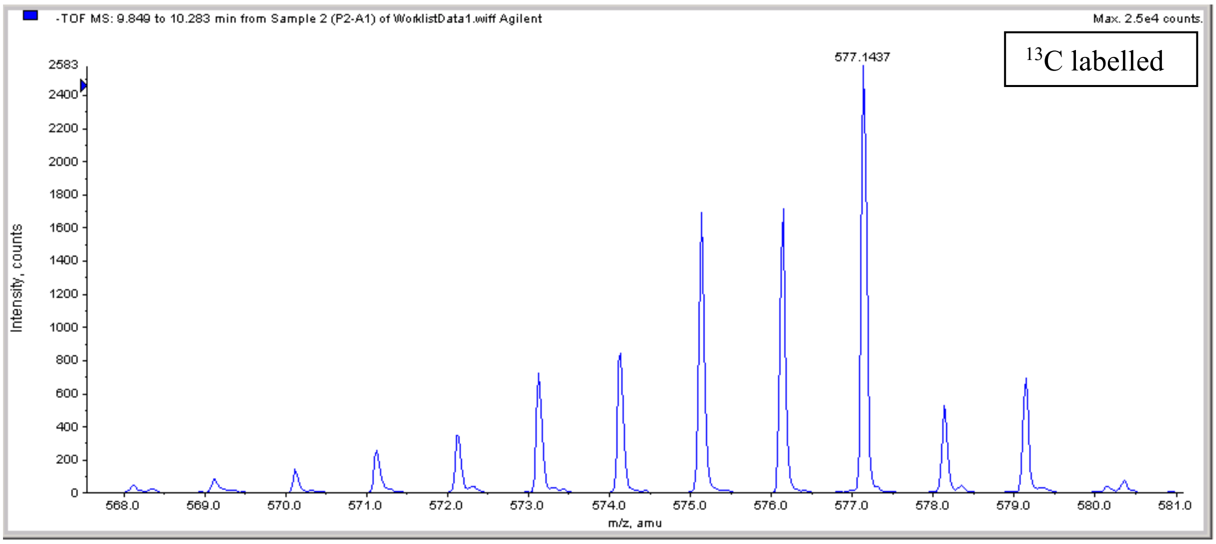
-TOF MS: 9.849 to 10.283 min from Sample 2 (P2-A1) of WorklistData1.wiff Agilent
Max. 2.5e4 counts.
^{13}C labelled
577.1437
Intensity, counts
2583
2400
2200
2000
1800
1600
1400
1200
1000
800
600
400
200
0
568.0 569.0 570.0 571.0 572.0 573.0 574.0 575.0 576.0 577.0 578.0 579.0 580.0 581.0
m/z, amu

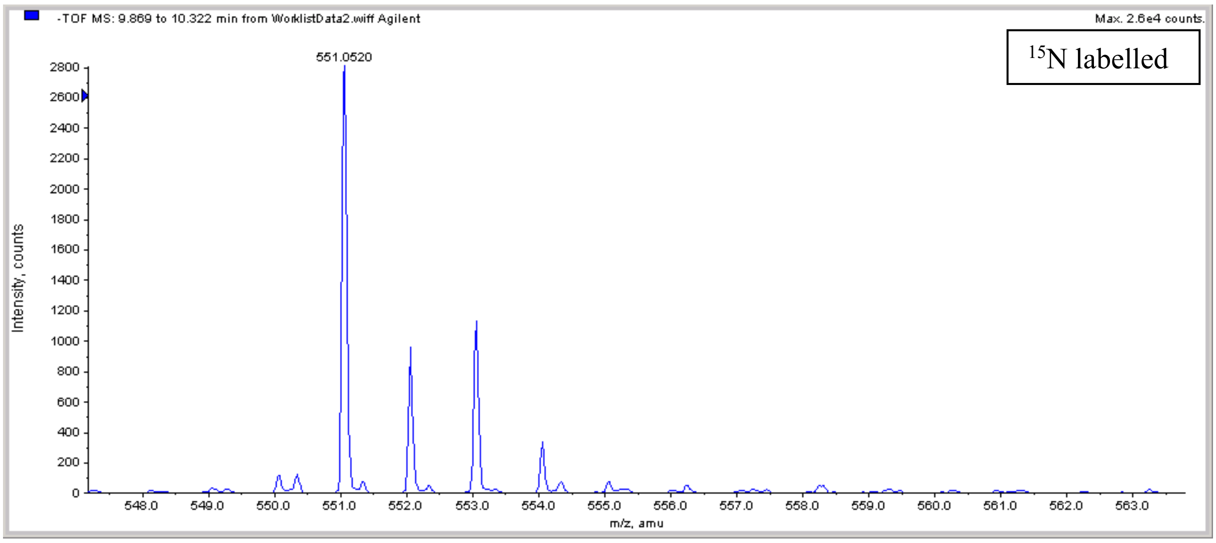
-TOF MS: 9.869 to 10.322 min from WorklistData2.wiff Agilent
Max. 2.6e4 counts.
^{15}N labelled
551.0520
Intensity, counts
2800
2600
2400
2200
2000
1800
1600
1400
1200
1000
800
600
400
200
0
548.0 549.0 550.0 551.0 552.0 553.0 554.0 555.0 556.0 557.0 558.0 559.0 560.0 561.0 562.0 563.0
m/z, amu

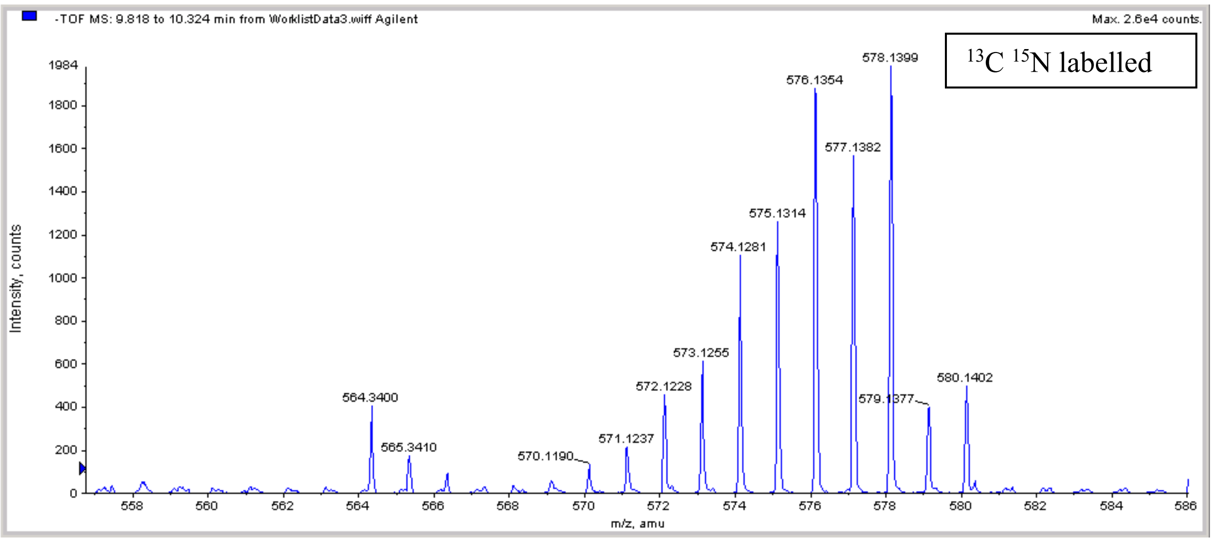

Fig. S2. LC-MS spectra from the stable isotope labelling experiment to determine the molecular formula of MBL-AB01. The observed masses in negative ionization mode (ESI-) of unlabelled, ^{13}C labelled, ^{15}N labelled, and ^{13}C and ^{15}N labelled MBL-AB01 were [m/z] = 550.0554 Da, [m/z] = 577.1437 Da, [m/z] = 551.0520 Da, and [m/z] = 578.1399 Da, respectively, demonstrating that MBL-AB01 has 27 carbon atoms and 1 nitrogen atom.

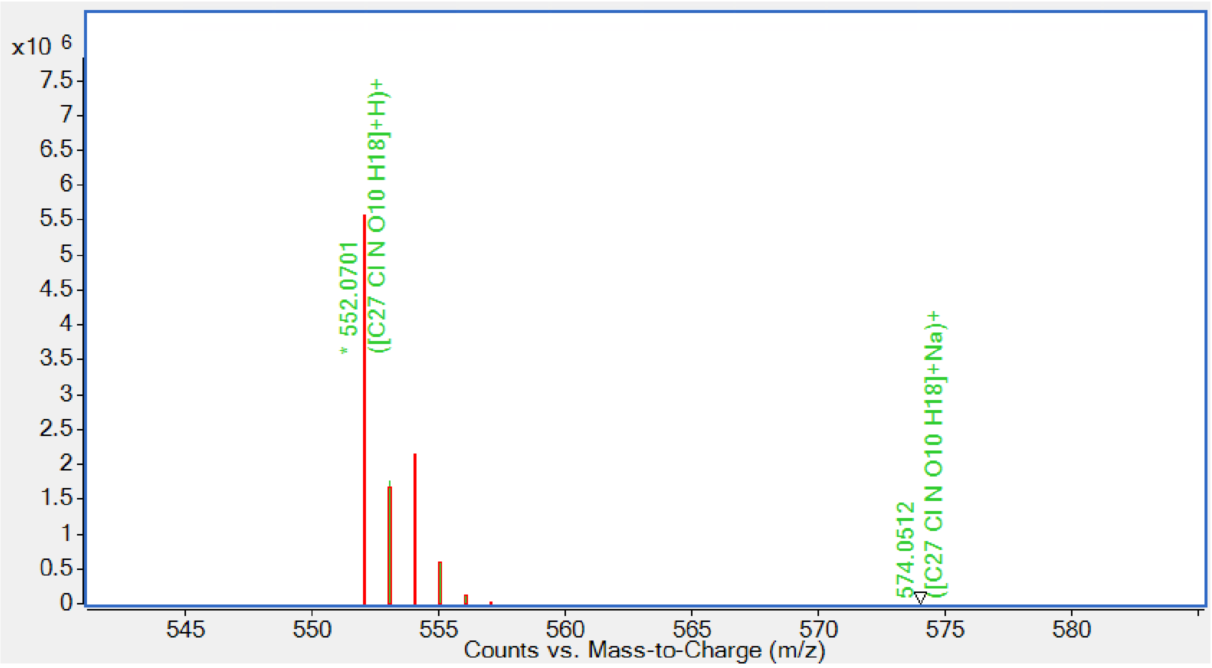

Fig. S3. MS spectrum of MBL-AB01 with matching molecular formula. The spectrum shows the [m/z] = 552.0701 corresponding to the positively charged MBL-AB01 and [m/z] = 574.0512 corresponding to the sodium adduct of MBL-AB01. The isotopic distribution indicated that the compound is halogenated. The proposed molecular formula $C_{27}H_{18}NO_{10}Cl$ matches the experimental isotopic distribution with a confidence score of 98.5. The measured monoisotopic mass aligns with the theoretical mass (1.4 ppm error) of the molecular formula. Molecular formula was generated by "Generate formula" in MassHunter10.0 (Agilent technologies) with element limits: C: 27-27, H: 0-120, O: 0-30, N:1-1, Cl: 0-1, Br: 0-1.

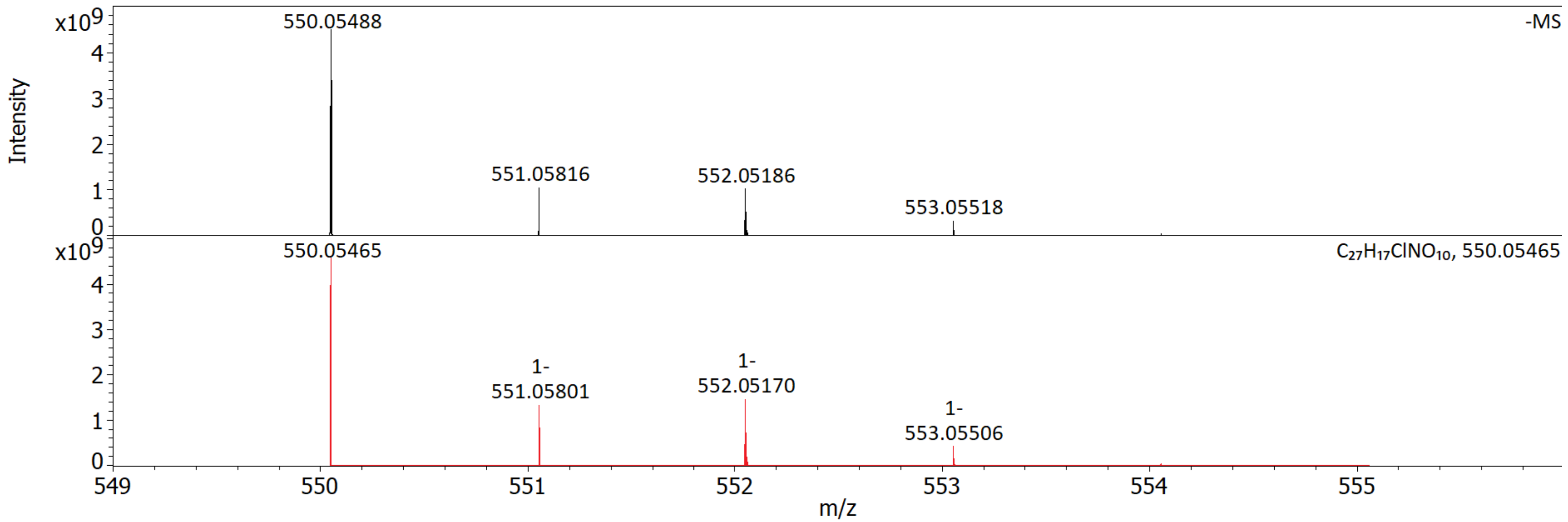

x10^9
550.05488
-MS
Intensity
551.05816
552.05186
553.05518
x10^9
550.05465
C27H17ClNO10, 550.05465
1-
551.05801
1-
552.05170
1-
553.05506
549
550
551
552
553
554
555
m/z

Panel A

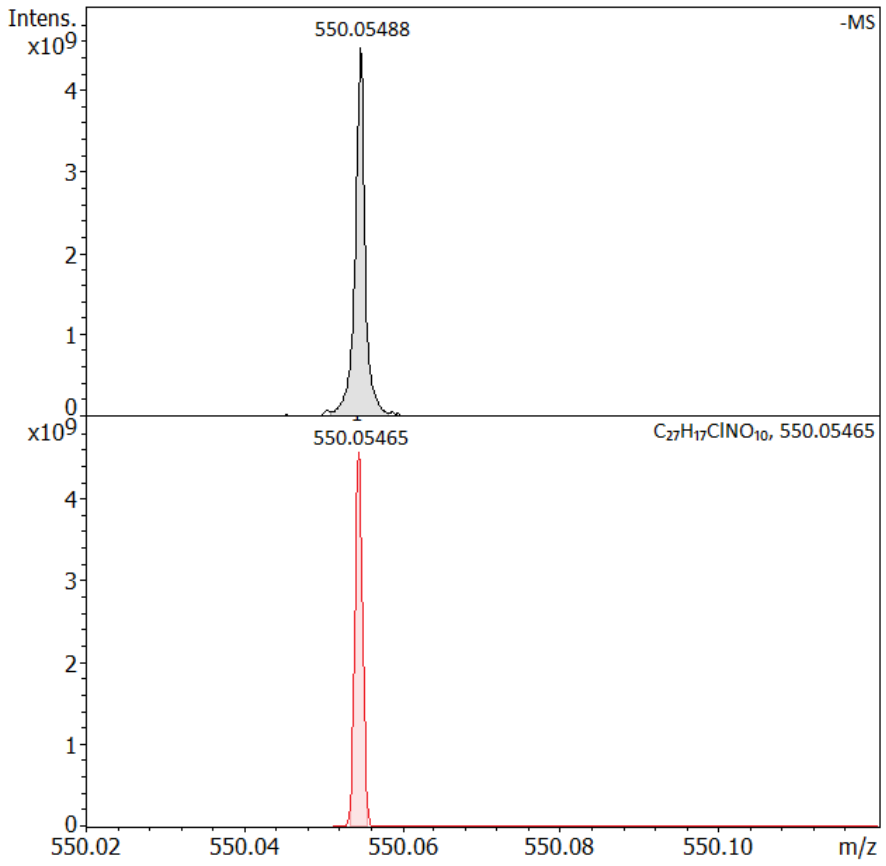

Panel B

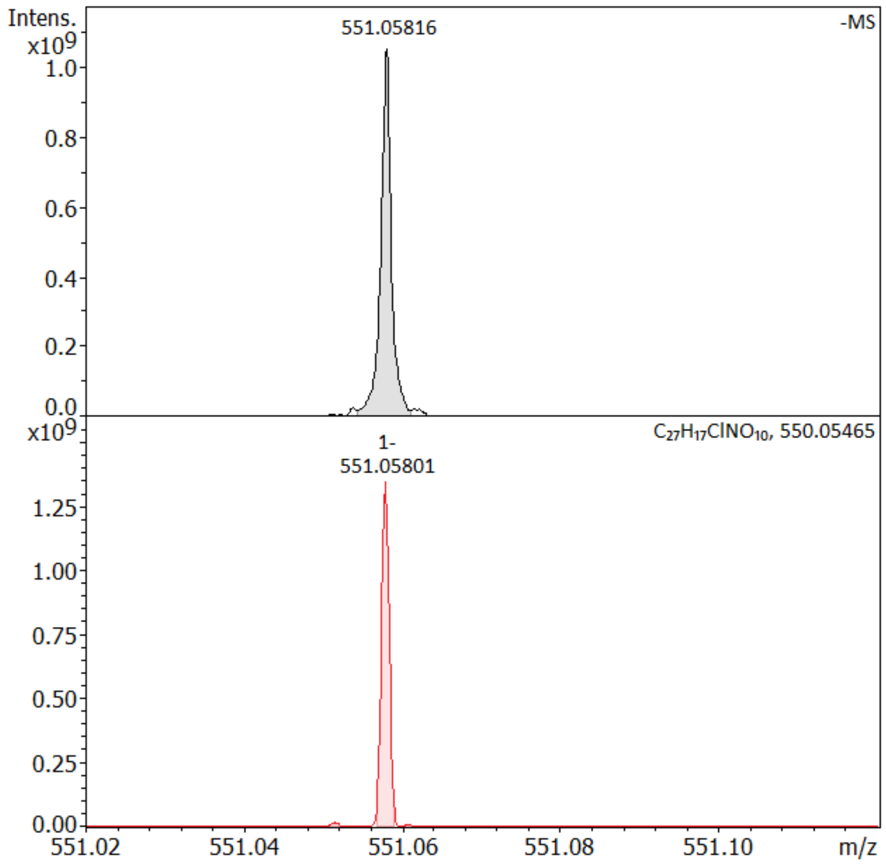

Panel C

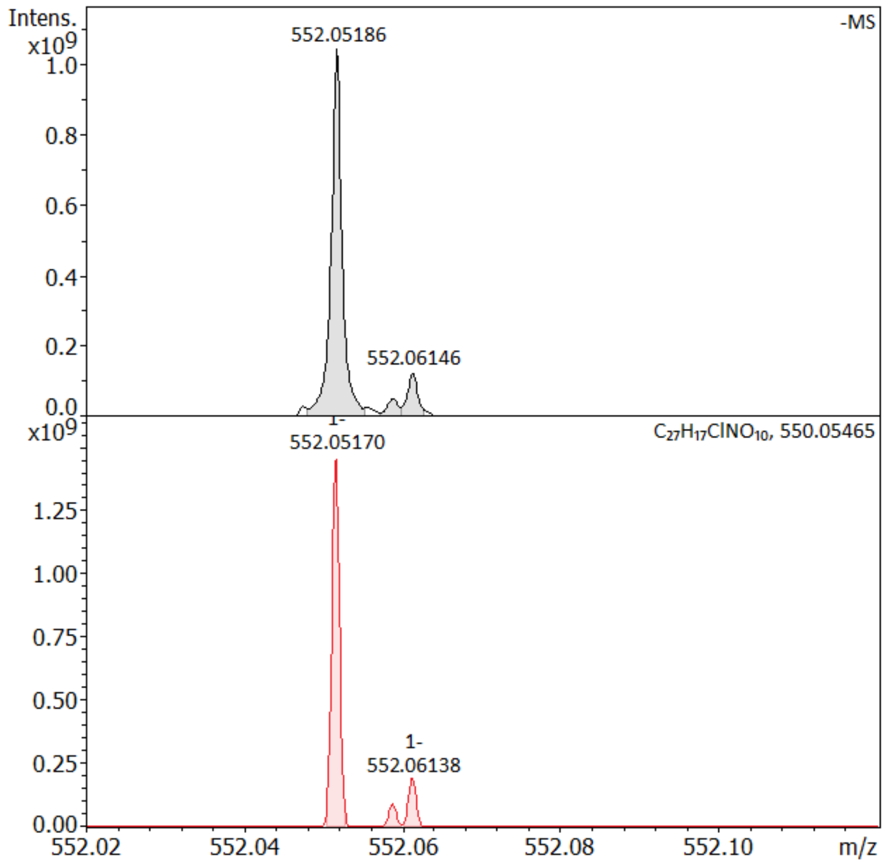

Panel D

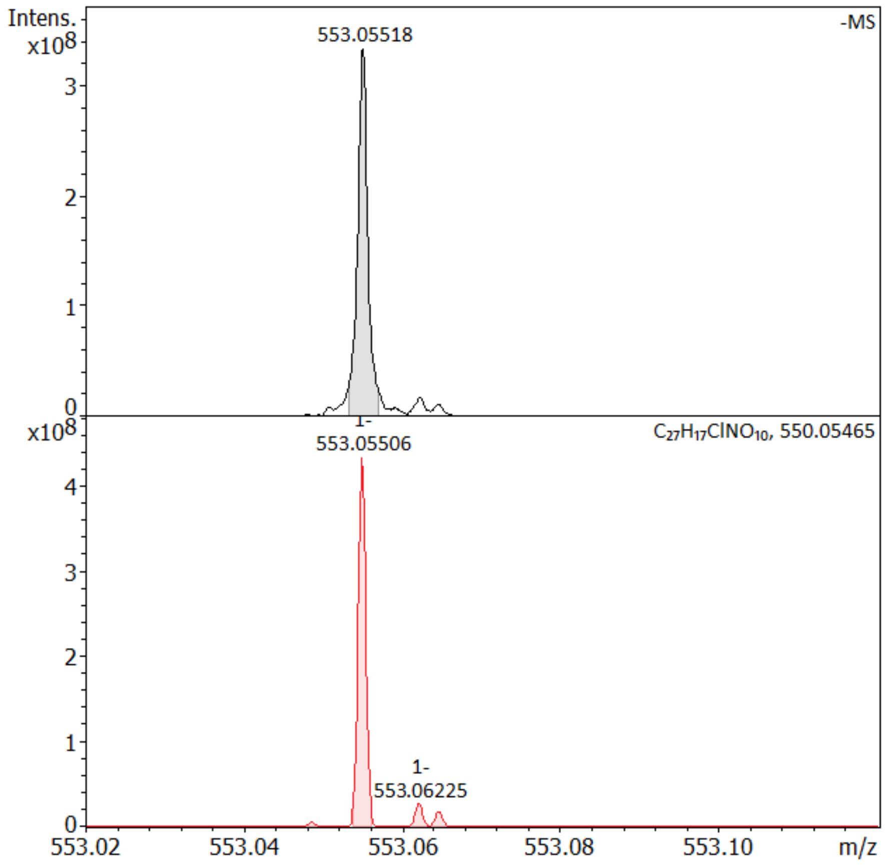

Panel E

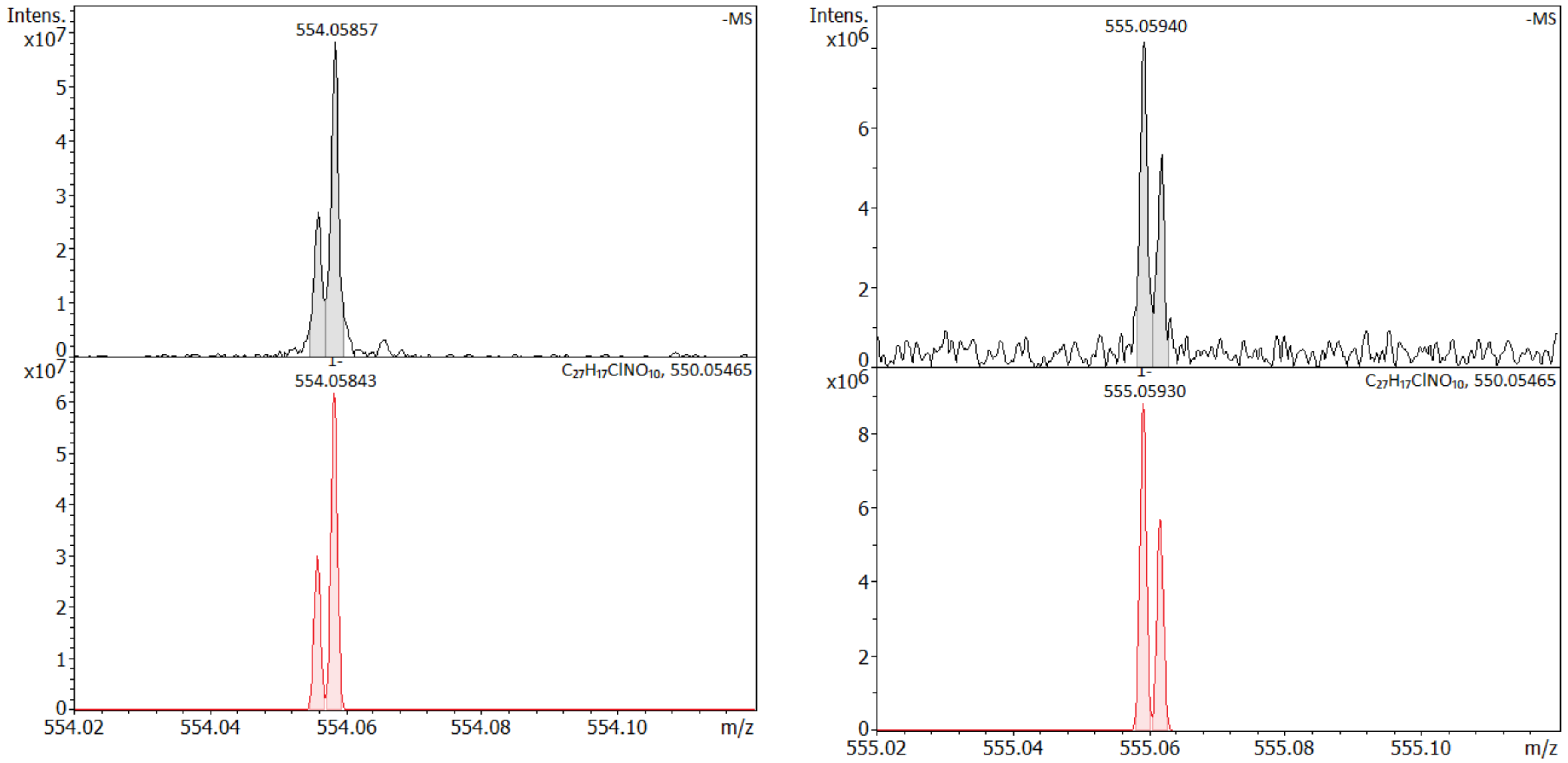

Panel F **Panel G**

Fig. S4. High resolution mass spectrum of MBL-AB01 collected on Bruker Solarix FT-ICR mass spectrometer in negative mode using electrospray ionization and direct infusion. **Panel A:** Full mass spectrum in mass range m/z 549 – 556. Isotope distribution shown for m/z 550.05488 (top of panel, black trace) compared with theoretical isotope pattern (bottom of panel, red trace) for the suggested ion formula $[M\text{-}H]^-$ $C_{27}H_{17}ClNO_{10}$. The suggested ion formula has score of 100, a m/z mass error of -0.4 ppm, and a msigma of 34. **Panel B-G**. Present zoomed-in views of the isotope

distributions, highlighting the signals for the molecular ion, $[M-H]^-$ with m/z = 550.05488 and for isotope peaks corresponding to from 1 to 5 atomic mass units higher than the $[M-H]^-$. The observed signals (top panel, black trace) correspond well to the theoretical isotope peaks (bottom panel, red trace), both in m/z for observed signals and their abundance.

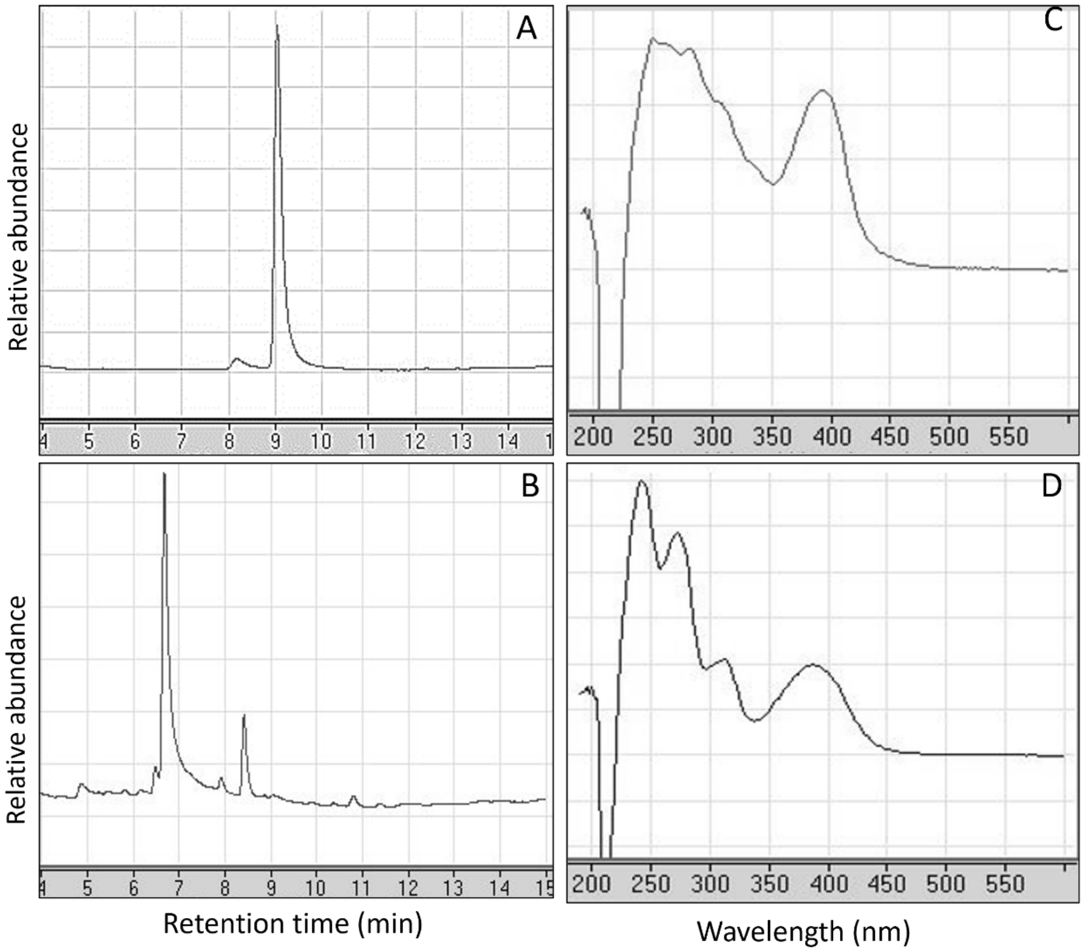

Fig. S5 LC-UV$_{395}$nm chromatogram of MBL-AB01 (A) and xantholipin (B) demonstrate that xantholipin elutes earlier from the HPLC column than MBL-AB01. UV spectra of MBL-AB01 (C) and xantholipin (D) show that the UV profiles of the two compounds are similar, but not identical.

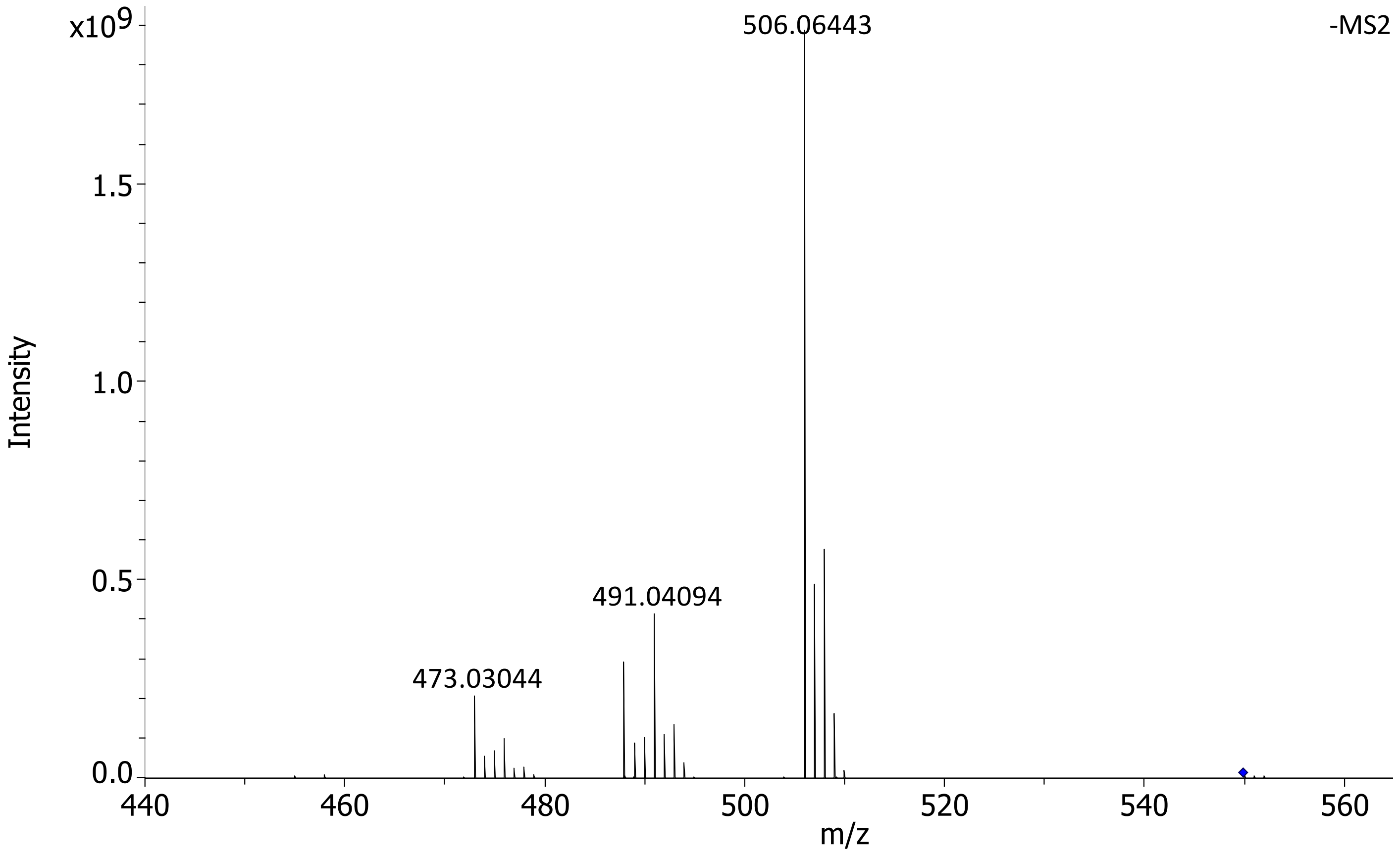

Fig. S6. High resolution mass spectrum of MBL-AB01 from collision induced fragmentation of $[M\text{-}H]^-$ ion (m/z=550.05488) at 20eV. The spectrum was collected on Bruker Solarix FT-ICR mass spectrometer in negative mode using direct infusion. The accurate mass data for the observed fragments from CID at 10 and 20 eV and suggested ion formula of fragments are given in Table S2.

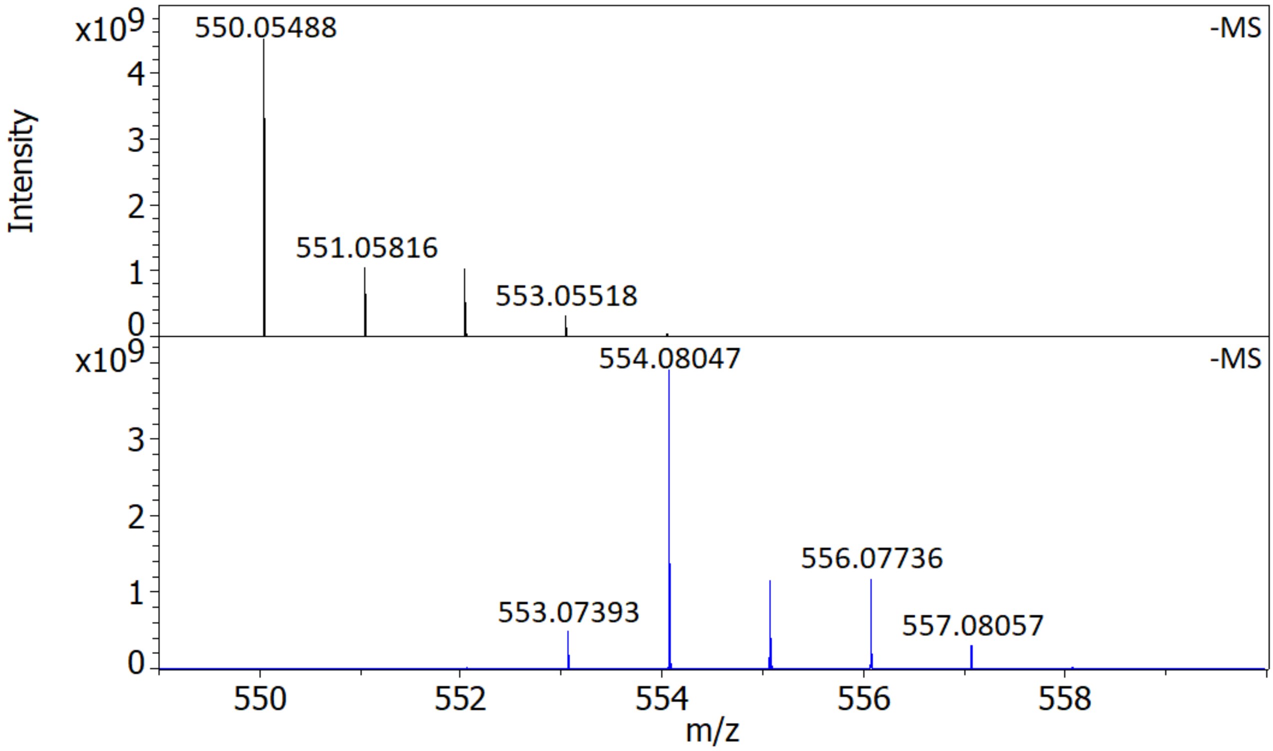

Fig. S7. High resolution mass spectrum of MBL-AB01 after hydrogen deuterium labelling. Spectra were collected on Bruker Solarix FT-ICR mass spectrometer in negative mode using direct infusion. Comparison of observed mass spectrum for MBL-AB01 (black trace) and MBL-AB01 after hydrogen deuterium exchange (blue trace). Main signal at m/z 554.0805 corresponds to ion formula [M-D]- with formula $C_{27}H_{13}ClD_4NO_{10}$ (mass deviation -1.3 ppm, score 100, msigma 8.3) with four deuterium. The suggested ion formulas of main observed ions and fragments resulting from collision induced fragmentation are given in Table S4.

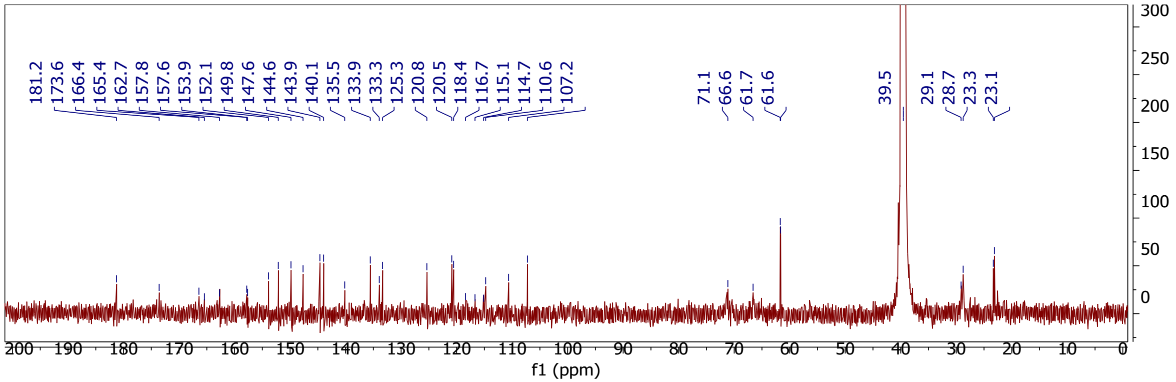

Fig. S8 1D ^{13}C NMR spectrum of MBL-AB01, 3.9 mg/mL in DMSO-d_6. The spectrum was acquired at 25 °C on an 800 MHz Bruker spectrometer equipped with a TCO probe.

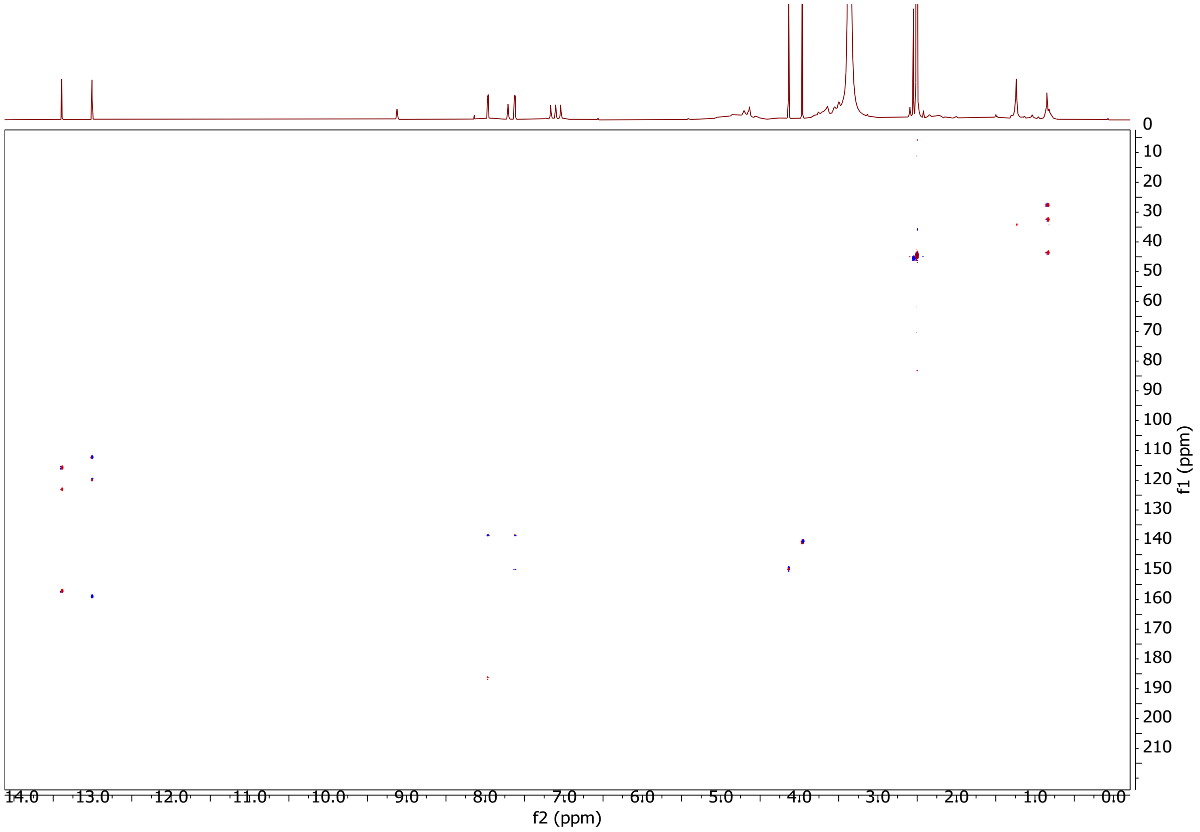

Fig. S9 2D ^{1}H-^{13}C HMBC spectrum of MBL-AB01, 3.9 mg/mL in DMSO-d_6. Spectrum is acquired at 25 °C on an 800 MHz Bruker spectrometer equipped with a TCO probe. Horizontal trace displays 1D ^{1}H spectrum.

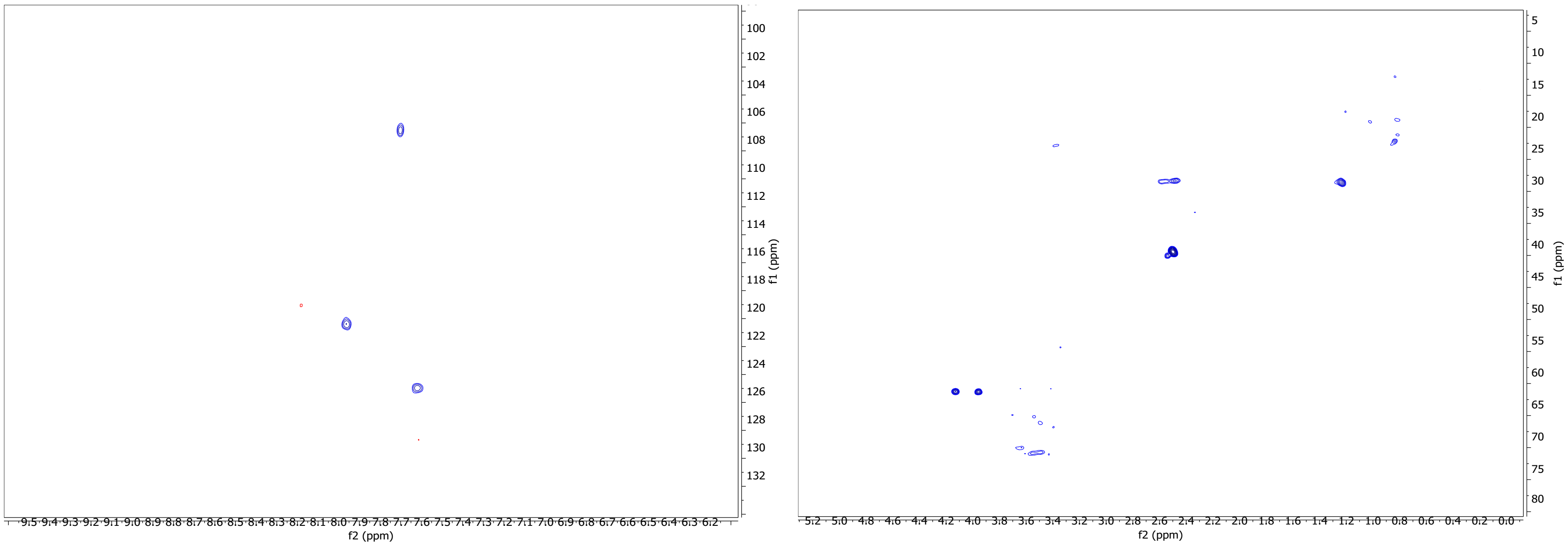

Fig. S10 2D ^{1}H-^{13}C HSQC spectra of MBL-AB01, 3.9 mg/mL in DMSO-d_6, aromatic signals region (left panel) and aliphatic signals region (right panel). Spectra are acquired at 25 °C on a 500 MHz Varian spectrometer equipped with a 5 mm $^{1}H/^{13}C/^{15}N$ triple resonance probe.

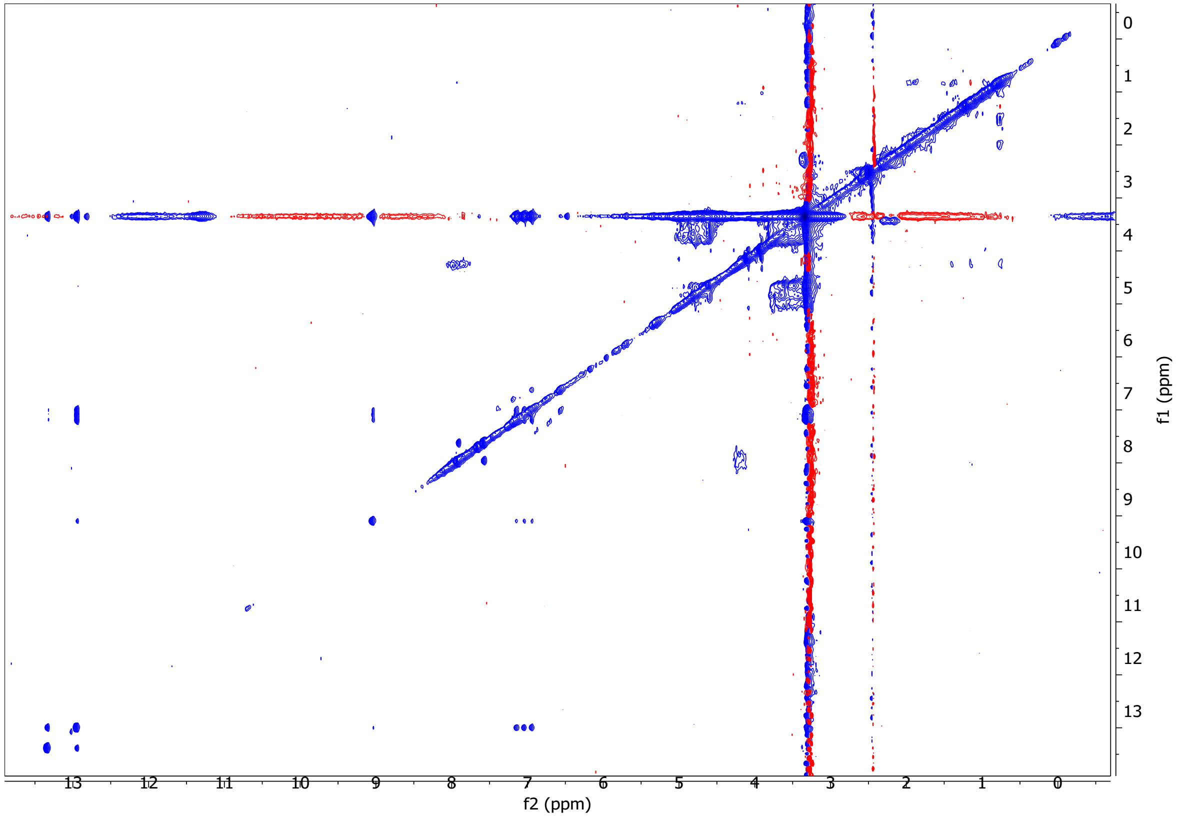

Fig. S11 2D ^1H NOESY spectrum (mixing time = 400 ms) of MBL-AB01, 3.9 mg/mL in DMSO-d_6. Spectrum is acquired at 25 °C on a 500 MHz Varian spectrometer equipped with a 5 mm $^1H/^{13}C/^{15}N$ triple resonance probe.

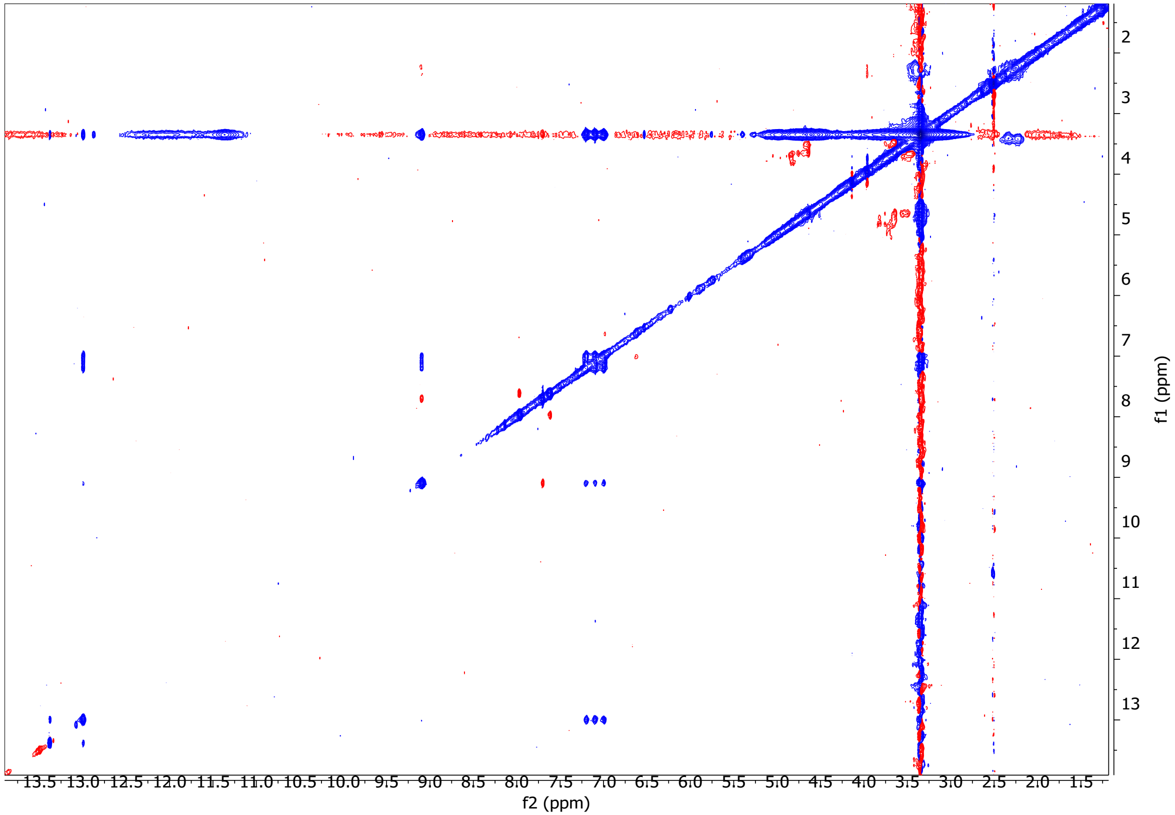

Fig. S12 2D ^{1}H adiabatic ROESY spectrum (mixing time = 200 ms) of MBL-AB01, 3.9 mg/mL in DMSO-d_6. Spectrum is acquired at 25 °C on a 500 MHz Varian spectrometer equipped with a 5 mm ^{1}H/^{13}C/^{15}N triple resonance probe.

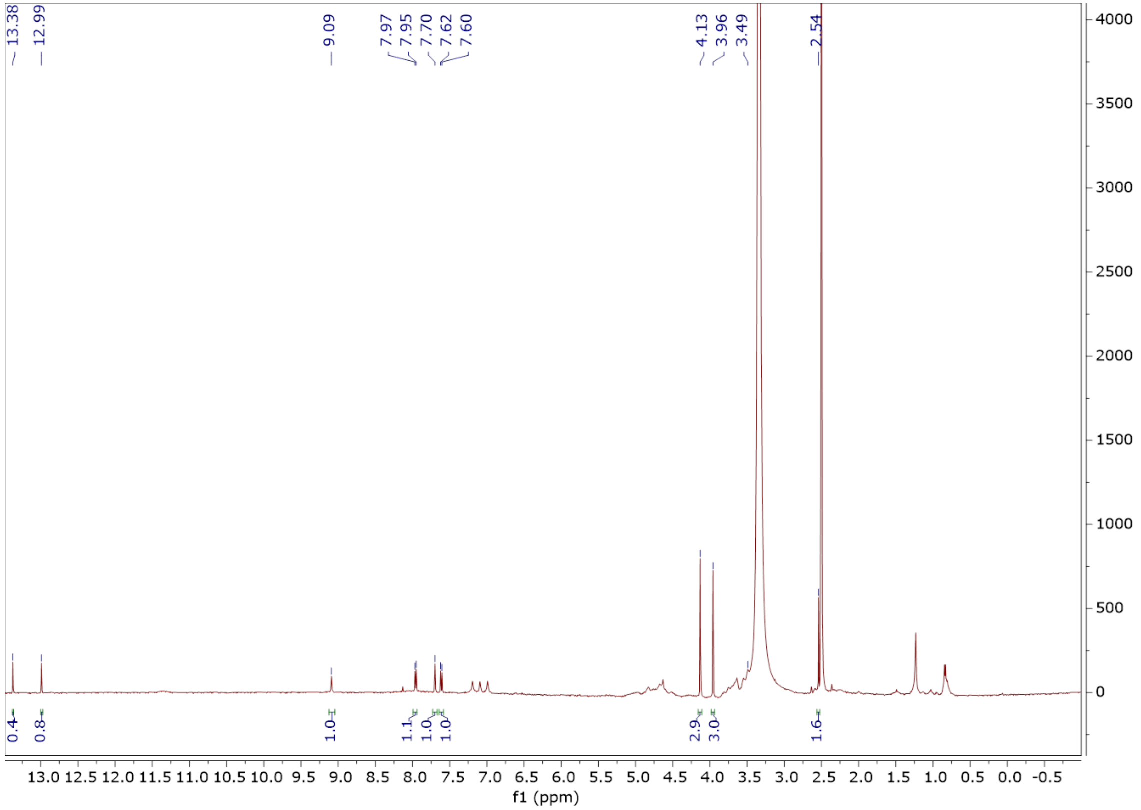

Fig. S13. 1D 1H NMR spectrum of MBL-AB01, 3.9 mg/mL in DMSO-d6. Spectrum is acquired at 25°C on a 500 MHz Varian Inova spectrometer equipped with a 5 mm 1H/13C/15N triple resonance probe.

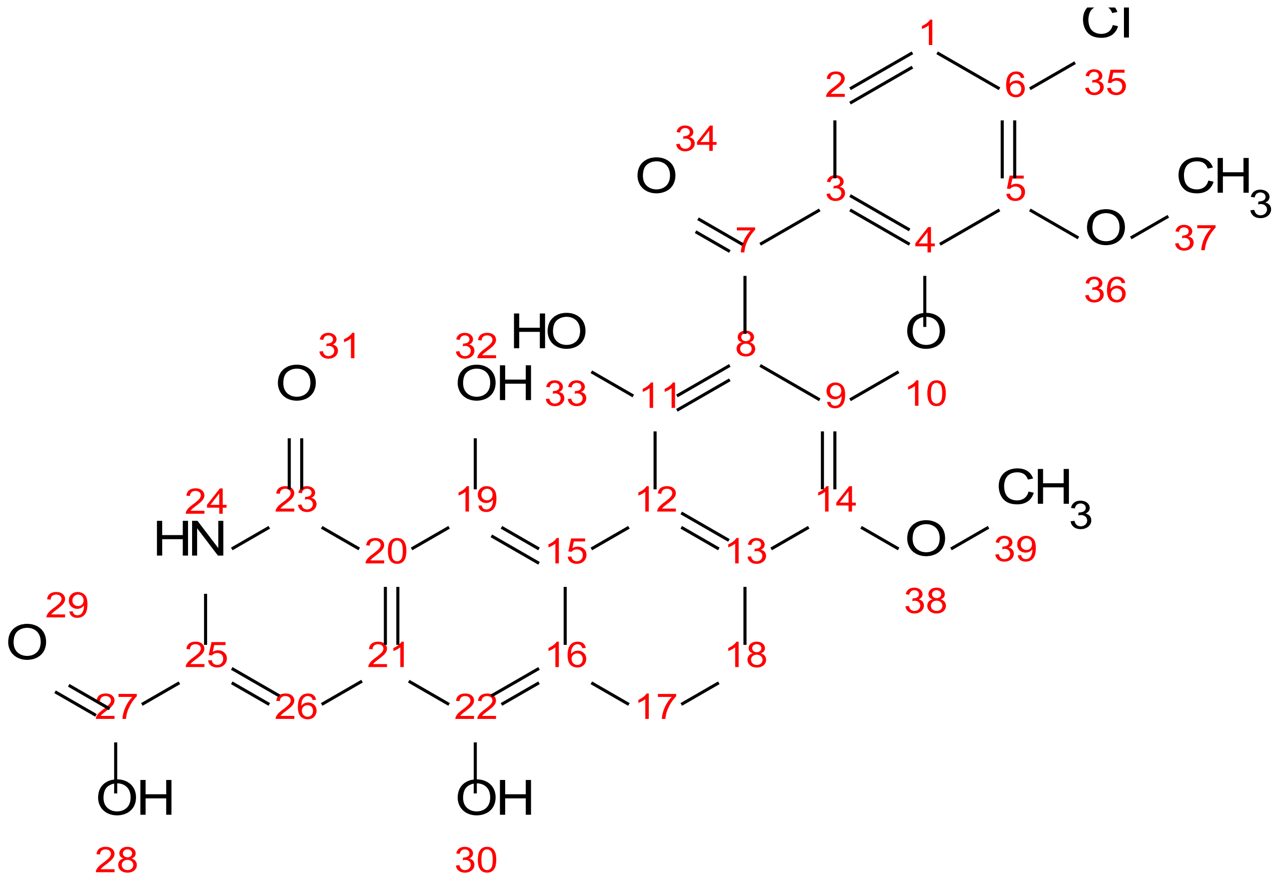

Fig. S14 The proposed structure of MBL-AB01 with atom numbers.

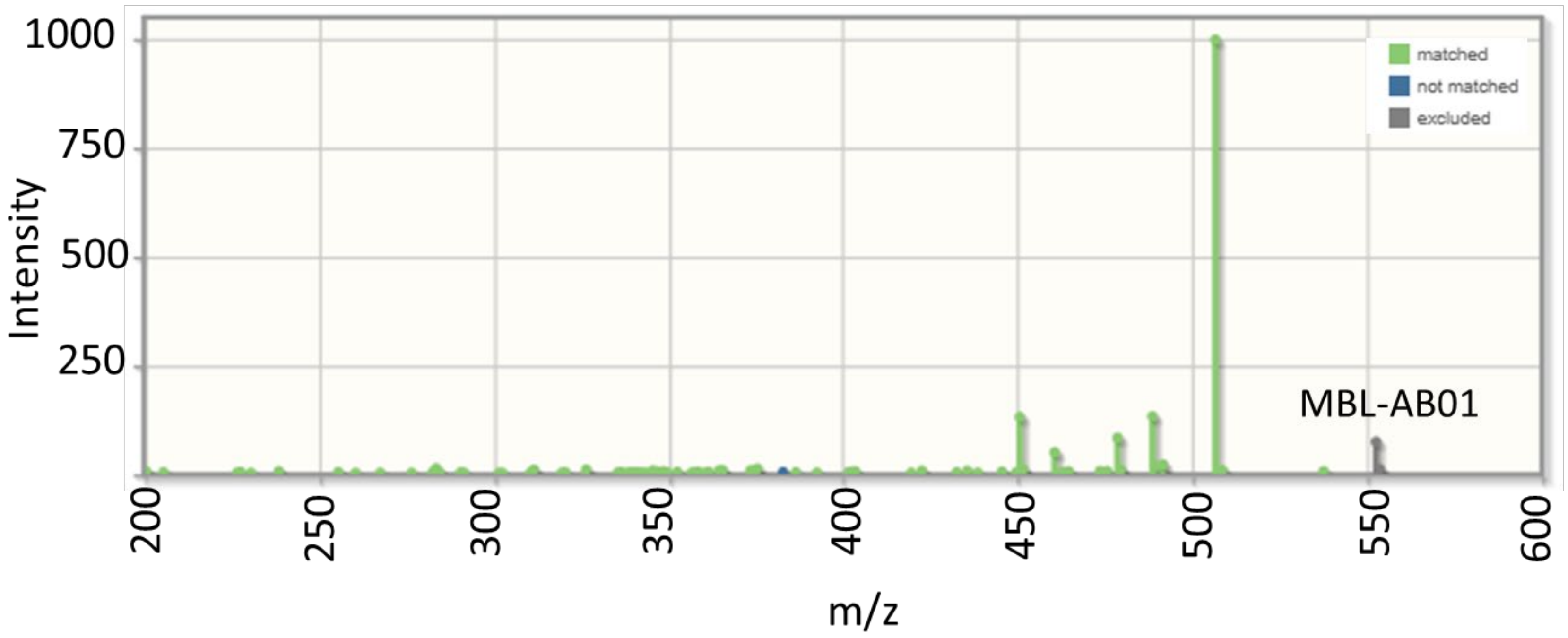

Fig. S15. The *in silico* fragmentation pattern of the proposed structure matches the observed MS/MS fragmentation data. 74 of the 75 fragments listed in Table S3 correspond to the *in silico* predicted fragments. The comparison was performed using MetFrag web (https://ipb-halle.github.io/MetFrag (Ruttkies et al. 2016)) with Mz abs=0.05, Mzppm=5, tree depth=2.

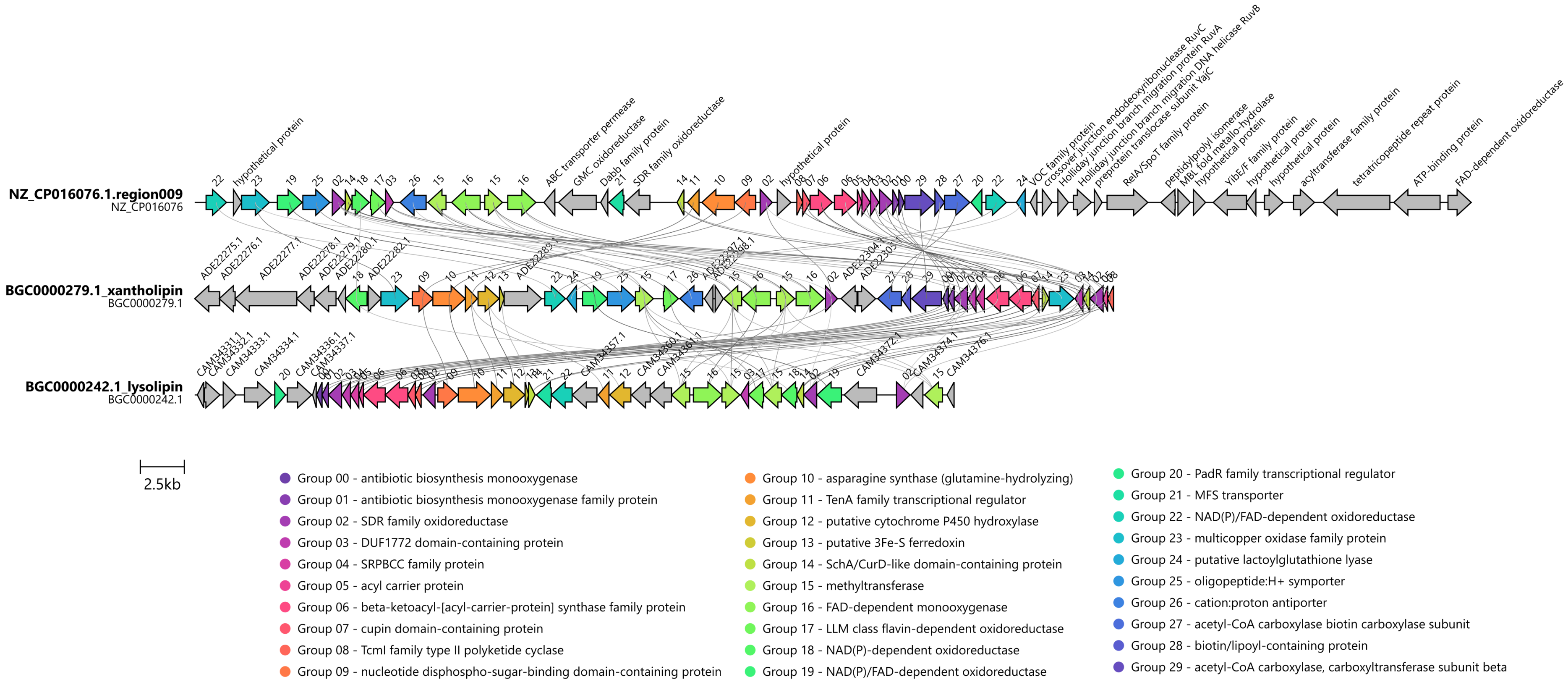

Fig. S16 Gene cluster comparison of the MBL-AB01 (NZ_CP016076.1.region009 obtained from https://antismash-db.secondarymetabolites.org/output/GCF_001941625.1/index.html#r1c9), xantholipin (BGC0000279.1 from https://mibig.secondarymetabolites.org/repository/BGC0000279.5/index.html#r1c1), and lysolipin (BGC0000242.1 from https://mibig.secondarymetabolites.org/repository/BGC0000242.5/index.html#r1c1) gene clusters created by clinker & clustermap.js (Afgan et

al. 2016) and further modified using Inkscape (https://inkscape.org). Thin lines link genes that are predicted to have similar functions with an identity threshold of 0.5. Genes within the same group (grouped by clinker) share the same color while those that are unique were depicted in grey color. In case of the MBL-AB01 BGC, the ungrouped genes were annotated with their predicted protein products while the annotation of ungrouped genes from the xantholipin and lysolipin BGCs can be looked up in their respective MiBIG entries. For the detailed comparison of genes among three clusters please refer to Table S7.

Table S1. Bioactivity (two independent parallels) of DMSO extracts of MP127-IG17 culture. A bioactivity score of 1 means that bioactivity was only detected in undiluted DMSO extract and a bioactivity score of 4 means activity was detected in both undiluted, 2-times and 4-times diluted DMSO extracts.

Indicator organism	Bioactivity score
M. luteus ATCC 9341	4
E. faecium CCUG 37832	1-2
C. Albans CCUG 39343	1

Table S2. High resolution mass spectrometry analysis of MBL-AB01. Summary of observed and theoretical m/z and ion formula assignments.

Experiment		Measured m/z	Intensity	Ion formula	Adduct	Charge	Theoretical m/z	Mass deviation (ppm)	Score	msigma	Loss*
		550.05488	4601492480	$C_{27}H_{17}ClNO_{10}$	[M-H]	-1	550.05465	-0.4	100	36.2	
Fragmentation of m/z 550.05	CID10	506.06442	1381310976	$C_{26}H_{17}ClNO_8$	[M-H]	-1	506.06482	0.8	100	10.7	CO_2
	CID20	473.03044	208026912	$C_{25}H_{12}ClNO_7$	[M-H]	-1	473.03078	0.7	100	6.5	$C_2H_5O_3$
		476.01750	100618192	$C_{24}H_{11}ClNO_8$	[M-H]	-1	476.01787	0.8	100	10.1	$C_3H_6O_2$
		488.05386	295739456	$C_{26}H_{15}ClNO_7$	[M-H]	-1	488.05425	0.8	100	12.8	CH_2O_3
		491.04094	413709600	$C_{25}H_{14}ClNO_8$	[M-H]	-1	491.04134	0.8	100	7.5	$C_2H_3O_2$
		506.06443	1898258176	$C_{26}H_{17}ClNO_8$	[M-H]	-1	506.06482	0.8	100	9.6	CO_2

msigma – SigmaFit presents the correlation between measured and theoretical isotopic pattern with lower value indicating better fit. CID: Collision induced fragmentation. * Loss compared to main ion signal for $[M-H]^-$.

Table S3. Main MS/MS fragments of the protonated MBL-AB01 ion [M+H] with m/z = 552.0670. Fragmentation was performed using a Bruker Impact II QTOF mass spectrometer. The resulting fragment ions were submitted to GNPS for dereplication and used in the *in silico* fragmentation analysis (see Fig. S15).

m/z	Intensity
506.0611661	51934
488.0511948	6968
450.0716902	6892
478.0663632	4412
552.0663357	3922
460.057215	2672
491.0383504	1224
489.0568083	1068
283.0610308	756
375.0037051	720
553.071998	692
451.076006	638
325.9948436	612
479.0669896	610
311.0556152	590
508.0622778	544
365.0207357	530
373.0149977	500
364.0109999	496
345.0195785	486
422.0777964	466
435.0479383	462
374.0165823	454
473.029324	432
475.0453761	428
200.0610824	410
238.063541	408
348.0161591	408
464.0515629	404
537.044554	396
346.020627	394
403.0237643	390
445.0347177	360
358.0247298	356
402.015091	352
361.0254373	344
352.0585024	340

227.0730636	328
336.0123385	310
382.3491542	300
255.0658321	294
462.0727829	292
386.0190765	290
463.0424066	290
432.063964	288
339.0484447	286
349.0289503	286
401.0050605	282
282.0517683	278
320.0194634	274
340.0594749	274
205.0522547	262
289.9938459	262
341.0211861	258
449.0644317	258
338.041076	250
357.0161074	250
310.0474475	248
392.0283018	234
438.0474156	232
284.0488584	230
291.0247212	230
347.014238	228
419.0159998	228
319.0139318	222
335.0279477	222
343.0382994	222
267.0648047	216
226.0651275	214
276.0209079	212
260.0297163	208
342.021675	206
230.0734096	204
302.0327335	204
356.0551025	204
301.0303048	200
360.0583273	200

Table S4. High resolution mass spectrometry analysis of MBL-AB01 after hydrogen-deuterium exchange. Summary of observed and theoretical m/z and ion formula assignments.

Experiment		Measured m/z	Intensity	Ion formula	Adduct	Charge	Theoretical m/z	Mass deviation (ppm)	Score	msigma
		553.07393	482871392	$C_{27}H_{14}ClD_3NO_{10}$	[M-D]	-1	553.07348	-0.8	100	>100
		554.08047	3980921600	$C_{27}H_{13}ClD_4NO_{10}$	[M-D]	-1	554.07975	-1.3	100	8.3
Fragmentation of 554	CID10	508.07695	64216168	$C_{26}H_{15}ClD_2NO_8$	[M-D]	-1	508.07737	0.8	100	>100
		509.08323	1247076864	$C_{26}H_{14}ClD_3NO_8$	[M-D]	-1	509.08365	0.8	100	11.9
		510.08952	336390112	$C_{26}H_{13}ClD_4NO_8$	[M-D]	-1	510.08993	0.8	100	22.7
	CID20	474.03674	10402388	$C_{25}H11ClDNO_7$	[M-D]	-1	474.03706	0.7	100	>100
		475.04296	235011920	$C_{25}H_{10}ClD_2NO_7$	[M-D]	-1	475.04333	0.8	100	7.7
		479.03620	35443636	$C_{24}H_8ClD_3NO_8$	[M-D]	-1	479.03670	1	100	27
		480.04251	71801080	$C_{24}H_7ClD_4NO_8$	[M-D]	-1	480.04298	1	100	9.8
		489.06012	16632280	$C_{26}H_{14}ClDNO_7$	[M-D]	-1	489.06053	0.8	100	>100
		490.06642	273960832	$C_{26}H_{13}ClD_2NO_7$	[M-D]	-1	490.06681	0.8	100	>100
		494.05979	149579232	$C_{25}H_{11}ClD_3NO_8$	[M-D]	-1	494.06017	0.8	100	97.4
		495.06608	451177344	$C_{25}H_{10}ClD_4NO_8$	[M-D]	-1	495.06645	0.7	100	13.9
		508.07697	99661040	$C_{26}H_{15}ClD_2NO_8$	[M-D]	-1	508.07737	0.8	100	>100
		509.08325	1902426752	$C_{26}H_{14}ClD_3NO_8$	[M-D]	-1	509.08365	0.8	100	10.3

msigma – SigmaFit presents the correlation between measured and theoretical isotopic pattern with lower value indicating better fit. CID: Collision induced fragmentation. HDX: Hydrogen deuterium exchange experiments.

Table S5. ^1H and ^{13}C chemical shifts for the proposed structure of MBL-AB01 in DMSO-d6 at 25 °C. *Atoms 17 and 18 might be interchanged, proton signals are broad and presented as an interval for atom 18. **Shift varies with pH. #Infered. The following ^{13}C signals could not be unambiguously assigned 116.7, 133.9, 143.9, 147.6, 166.4, 173.6 ppm (given as not determined).

#	δ_H [ppm]	δ_C [ppm]	^1H-^{13}C short range correlation (HSQC) observed	^1H-^{13}C long range correlation (HMBC) observed	ROE correlation
**1**	7.60 (d, J = 10 Hz, 1H)	125.3	Yes	H-1 → C-5, C-6	
**2**	7.96 (d, J = 10 Hz, 1H)	120.8	Yes	H-2 → C-6, C-7	
**3**	-	120.5	-	-	
**4**	-	149.8	-	-	
**5**	-	144.6	-	-	
**6**	-	133.3	-	-	
**7**	-	181.2	-	-	
**8**	-	107.2	-	-	
**9**	-	not determined	-	-	
**11**	-	153.9	-	-	
**12**	-	114.7	-	-	
**13**	-	not determined	-	-	
**14**	-	135.5	-	-	
**15**	-	118.4	-	-	
**16**	-	not determined	-	-	
**17***	3.40 (bm, 2H#)	23	Yes	-	
**18***	2.4-2.7 (bm, 2H#)	28	Yes	-	
**19**	-	152.1	-	-	
**20**	-	110.6	-	-	
**21**	-	not determined	-	-	

**22**	-	not determined	-	-	
**23**	-	not determined	-	-	
**24**	-	-	-	-	
**25**	-	140.1	-	-	
**26****	7.73/7.46 (s, 1H)	107.2/101.5	Yes	H-26 → C-20, C-27	H-26 → H-30
**27**	-	162.7	-	-	
**28**	Not observed	-	-	-	
**30**	9.07 (s, 1H)	-	-		H-30 → H-26
**32**	13.38 (s, 1H)	-	-	H-32 → C-15, C-19, C-20	
**33**	12.99 (s, 1H)	-	-	H-33 → C-8, C-11, C-12	
**37**	4.13 (s, 3H)	61.6	Yes	H-37 → C-5	
**39**	3.95 (s, 3H)	61.6	Yes	H-39 → C-14	

Table S6. ^{1}H and ^{13}C chemical shifts for MBL-AB01 in DMSO-d_6 at 25 °C, presented alongside the corresponding values reported for the corresponding atom positions in Xantholipin (Terui et al. 2003) in $CDCl_3$ at 25 °C. Atom numbering refers to Fig. S14.

#	δ_H [ppm]	δ_C [ppm]	δ_H Xantholipin [ppm]	δ_C Xantholipin [ppm]
**1**	7.60	125.3	7.52	125.5
**2**	7.96	120.8	7.78	120.5
**3**	-	120.5	-	120.2
**4**	-	149.8	-	150.0
**5**	-	144.5	-	144.5
**6**	-	133.0	-	133.4
**7**	-	181.2	-	181.3
**37**	4.13	61.6	4.01	61.4

Table S7: Genes found in the chromosomal region believed to be responsible for production of MBL-AB01. Locus_tags, region, and product descriptions refer to the highly similar reference genome for *Actinoalloteichus fjordicus* strain ADI127-7 (Genbank: NZ_CP016076). The initial gene cluster was predicted by running antiSMASH on a draft assembly made from Illumina MiSeq sequencing reads. The list was then supplemented by neighbouring genes that showed high sequence similarity to the xantholipin (xan) and/or lysolipin (llp) reference clusters.

**Published genome (NZ_CP016076)**			**Best match Xantholipin (aa)**		**Best match Lysolipin (aa)**		**Group shown on Fig. S16**
**Locus_tag**	**Region**	**Product**	**Gene**	**Aa Identity/ Simiarity/ Overlap (%)**	**Gene**	**Aa Identity/ Simiarity/ Overlap (%)**	
**UA74_RS10920**	2423323 - 2422130	cytochrome P450	xanO2	42.5 / 57 / 97.5	llpOVI	37.2 / 53.4 / 90.7	
**UA74_RS10925**	2424415 - 2423483	oxidoreductase	xanS2	59.6 / 70.4 / 97.8	llpU	40.0 / 47.8 / 26.1	
**UA74_RS10930**	2425264 - 2426427	NAD(P)/FAD-dependent oxidoreductase	xanO3	55.1 / 68.8 / 97.2	llpOV	35.0 / 46.0 / 64.6	22
**UA74_RS10935**	2426819 - 2427283	hypothetical protein	xanJ	42.8 / 58.6 / 58.3	-		
**UA74_RS10940**	2427280 - 2428866	multicopper oxidase family protein	xanP	61.3 / 72.9 / 92.4	-		23
**UA74_RS10945**	2429315 - 2430730	NAD(P)/FAD-dependent oxidoreductase	xanH	72.8 / 83.9 / 98.9	llpH	70.4 / 83.4 / 97.7	19
**UA74_RS10950**	2430767 - 2432299	oligopeptide:H+ symporter	xanQ	57.5 / 72.1 / 91.0	-		25
**UA74_RS10955**	2432441 - 2433178	SDR family oxidoreductase	xanZ4	62.5 / 77.1 / 97.6	llpZIII	71.5 / 81.5 / 90.2	02
**UA74_RS10960**	2433178 - 2433558	SchA/CurD-like domain-containing protein	xanV	74.0 / 87.8 / 97.6	llpV	77.2 / 90.2 / 97.6	14
**UA74_RS10965**	2433564 - 2434547	NAD(P)-dependent oxidoreductase	xanS1	57.9 / 73.6 / 59.5	llpS	59.6 / 75.0 / 73.4	18
**UA74_RS10970**	2434609 - 2435472	LLM class flavin-dependent oxidoreductase	xanZ1	60.8 / 75.9 / 100.4	llpZII	59.7 / 74.0 / 98.6	17
**UA74_RS10975**	2435469 - 2435924	DUF1772 domain-containing protein	xanO10	62.7 / 76.3 / 79.1	llpQ	65.7 / 73.3 / 68.9	03
**UA74_RS10980**	2437776 - 2436295	cation:proton antiporter	xanN	39.7 / 56.9 / 93.7	-		26
**UA74_RS10985**	2438909 - 2437878	methyltransferase	xanM2	64.0 / 77.8 / 97.4	llpMIII	61.2 / 73.1 / 97.7	15
**UA74_RS10990**	2440851 - 2439226	FAD-dependent monooxygenase	xanO4	74.2 / 85.2 / 95.4	llpOVIII	75.8 / 84.9 / 91.3	16
**UA74_RS10995**	2441110 - 2442117	methyltransferase	xanM3	66.1 / 78.6 / 97.1	llpMII	66.1 / 80.3 / 98.5	15

**UA74_RS11000**	2442420 - 2444018	FAD-dependent monooxygenase	xanO5	60.7 / 75.0 / 97.4	llpOVIII	38.5 / 52.6 / 92.5	16
**UA74_RS11005**	2445096 -2444482	ABC transporter permease	xanJ	42.8 / 58.6 / 58.3			
**UA74_RS11010**	2447454 - 2445307	GMC oxidoreductase					
**UA74_RS11015**	2448113 - 2447706	Dabb family protein					
**UA74_RS11020**	2449025 - 2448171	SDR family oxidoreductase			llpL	33.0 / 48.5 / 92.3	
**UA74_RS11025**	2450506 - 2449088	MFS transporter			llpN	26.4 / 51.5 / 33.3	21
**UA74_RS11035**	2452439 - 2452050	SchA/CurD-like domain-containing protein	xanT	67.2 / 80.7 / 93.0	llpT	77.7 / 85.1 / 93.8	14
**UA74_RS11040**	2453301 - 2452636	TenA family transcriptional regulator	xanR1	67.6 / 75.7 / 97.4	llpRII	60.4 / 69.8 / 100.5	11
**UA74_RS11045**	2455304 - 2453460	asparagine synthase (glutamine-hydrolyzing)	xanA	68.6 / 80.3 / 100	llpA	67.1 / 77.7 / 100	10
**UA74_RS11050**	2456520 - 2455351	nucleotide disphospho-sugar-binding domain-containing protein	xanG	56.9 / 71.8 / 93.6	llpG	56.4 / 69.8 / 91	09
**UA74_RS11055**	2456778 - 2457461	SDR family NAD(P)-dependent oxidoreductase	xanS2	59.6 / 70.4 / 97.8	llpU	70.4 / 77.1 / 98.2	02
**UA74_RS11060**	2457748 - 2458521	hypothetical protein					
**UA74_RS11065**	2458861 - 2459196	TcmI family type II polyketide cyclase	xanC3	67.3 / 82.7 / 98.2	llpCIII	80 / 88.2 / 99.1	08
**UA74_RS11070**	2459193 - 2459621	cupin domain-containing protein	xanC2	69.1 / 80.6 / 96.5	llpCII	72.7 / 82.7 / 97.9	07
**UA74_RS11075**	2459618 - 2460886	beta-ketoacyl-[acyl-carrier-protein] synthase family protein	xanF	81.2 / 90.7 / 98.6	llpF	78.5 / 87.6 / 99.1	06
**UA74_RS11080**	2461000 - 2462241	ketosynthase chain-length factor	xanE	72.2 / 80.5 / 94.2	llpE	71.6 / 81.4 / 93	06
**UA74_RS11085**	2462300 - 2462560	acyl carrier protein	xanD	44.2 / 64.0 / 98.8	llpD	50.0 / 65.1 / 100	05
**UA74_RS11090**	2462565 - 2463032	SRPBCC family protein	xanC1	67.8 / 80.5 / 96.1	llpCI	59.3 / 77.9 / 93.6	04
**UA74_RS11095**	2463061 - 2463522	DUF1772 domain-containing protein	xanO8	66.7 / 77.2 / 74.5	llpB	58.8 / 71.9 / 74.5	03
**UA74_RS11100**	2463560 - 2464312	SDR family NAD(P)-dependent oxidoreductase	xanZ3	72.4 / 85.6 / 100	llpZI	76.4 / 85.6 / 100	02
**UA74_RS11105**	2464312 - 2464662	antibiotic biosynthesis monooxygenase family protein	xanO7	58.4 / 75.3 / 87.0	llpOIII	73.6 / 84.6 / 94.8	01
**UA74_RS11110**	2464659 - 2464961	antibiotic biosynthesis monooxygenase	xanO6	59.2 / 70.9 / 97.1	llpOII	51.5 / 63.1 / 103	00

**UA74_RS11115**	2465003 - 2466718	acetyl-CoA carboxylase, carboxyltransferase subunit beta	xanB3	64.9 / 75.8 / 95.4			29
**UA74_RS11120**	2466715 - 2467260	biotin/lipoyl-containing protein	xanB2	49.4 / 57.6 / 96.0			28
**UA74_RS11125**	2467266 - 2468696	acetyl-CoA carboxylase biotin carboxylase subunit	xanB1	72.0 / 82.8 / 98.9			27
**UA74_RS11130**	2469384 - 2468764	PadR family transcriptional regulator			llpRI	39.3 / 55.6 / 83.0	20
**UA74_RS11135**	2469615 - 2470778	NAD(P)/FAD-dependent oxidoreductase	xanO3	55.1 / 68.8 / 97.2	llpOV	29.1 / 40.9 / 82.5	22

Table S8. The composition of the eleven production media tested is based on the PLM6 formulation (Engelhardt et al. 2010). Each medium was supplemented with 0.5× artificial seawater to support marine microbial growth.

**Component**	**PML6_MOD3**	**PML6_MOD5**	**PML6_MOD6**	**PML6_MOD7**	**PML6_MOD8**	**PML6_MOD9**	**PML6_MOD10**	**PML6_MOD11**	**PML6_MOD12**	**PML6_MOD16**	**PML6_MOD17**
Soluble starch (Sigma)	30.0									30.0	30.0
Soluble starch (Difco)		50.0	50.0	50.0	50.0	100.0	50.0	50.0	50.0		
Yeast extract	2.0	2.0	2.0	2.0	2.0	4.0	4.0	2.0	2.0	4.0	2.0
Pepton	2.0	2.0	2.0	2.0	2.0	4.0	2.0	2.0	2.0	4.0	2.0
NaCl									0.2		
Na-glutamate			7.0								
NH_4Cl				2.0							
$NaNO_3$					3.2						
Cornsteep liquid	2.5	2.5	2.5	2.5	2.5	2.5	2.5	2.5	2.5	2.5	5.0
Soy flour								30.0			
MOPS			21.0	21.0	21.0						
$CaCO_3$	3.0	3.0	3.0	3.0	3.0	6.0	3.0	3.0	3.0	6.0	3.0

References

Afgan, Enis, Dannon Baker, Marius van den Beek, et al. 2016. “The Galaxy Platform for Accessible, Reproducible and Collaborative Biomedical Analyses: 2016 Update.” *Nucleic Acids Research* 44 (W1): W3–10. https://doi.org/10.1093/nar/gkw343.

Engelhardt, K., K. F. Degnes, M. Kemmler, et al. 2010. “Production of a New Thiopeptide Antibiotic, TP-1161, by a Marine Nocardiopsis Species.” *Applied and Environmental Microbiology* 76 (15): 4969–76. https://doi.org/10.1128/Aem.00741-10.

Ruttkies, Christoph, Emma L. Schymanski, Sebastian Wolf, Juliane Hollender, and Steffen Neumann. 2016. “MetFrag Relaunched: Incorporating Strategies beyond in Silico Fragmentation.” *Journal of Cheminformatics* 8 (1): 3. https://doi.org/10.1186/s13321-016-0115-9.

Terui, Yuichi, Chu Yiwen, Li Jun-ying, et al. 2003. “Xantholipin, a Novel Inhibitor of HSP47 Gene Expression Produced by Streptomyces Sp.” *Tetrahedron Letters* 44 (29): 5427–30. https://doi.org/10.1016/S0040-4039(03)01318-2.
